# New Succinimides with Potent Anticancer Activity: Synthesis, Activation of Stress Signaling Pathways and Characterization of Apoptosis in Leukemia and Cervical Cancer Cells

**DOI:** 10.3390/ijms22094318

**Published:** 2021-04-21

**Authors:** Marcin Cieślak, Mariola Napiórkowska, Julia Kaźmierczak-Barańska, Karolina Królewska-Golińska, Anna Hawrył, Iwona Wybrańska, Barbara Nawrot

**Affiliations:** 1 Centre of Molecular and Macromolecular Studies, Department of Bioorganic Chemistry, Polish Academy of Sciences, 112 Sienkiewicza, 90-363 Lodz, Poland; juliakazmierczak@tlen.pl (J.K.-B.); kkrolews@cbmm.lodz.pl (K.K.-G.); bnawrot@cbmm.lodz.pl (B.N.); 2 Department of Biochemistry, Faculty of Medicine, Medical University of Warsaw, 1 Banacha, 02-097 Warsaw, Poland; 3 Department of Inorganic Chemistry, Faculty of Pharmacy, Medical University of Lublin, Chodźki 4a, 20-093 Lublin, Poland; anna.hawryl@umlub.pl; 4 Clinical Biochemistry, Department of Genetics and Nutrigenomics, Faculty of Medicine, Jagiellonian University Medical College, 15a Kopernika, 31-501 Kraków, Poland; mbwybran@cyf-kr.edu.pl

**Keywords:** dicarboximides, chemical synthesis, cytotoxicity, apoptosis, kinases, anticancer, gene profiling, SAR

## Abstract

Based on previously identified dicarboximides with significant anticancer and immunomodulatory activities, a series of 26 new derivatives were designed and synthesized by the Diels–Alder reaction between appropriate diene and maleimide or hydroxymaleimide moieties. The resulting imides were functionalized with alkanolamine or alkylamine side chains and subsequently converted to their hydrochlorides. The structures of the obtained compounds were confirmed by 1H and 13C NMR and by ESI MS spectral analysis. Their cytotoxicity was evaluated in human leukemia (K562, MOLT4), cervical cancer (HeLa), and normal endothelial cells (HUVEC). The majority of derivatives exhibited high to moderate cytotoxicity and induced apoptosis in K562 cells. Microarray gene profiling demonstrated upregulation of proapoptotic genes involved in receptor-mediated and mitochondrial cell death pathways as well as antiapoptotic genes involved in NF-kB signaling. Selected dicarboximides activated JNK and p38 kinases in leukemia cells, suggesting that MAPKs may be involved in the regulation of apoptosis. The tested dicarboximides bind to DNA as assessed by a plasmid DNA cleavage protection assay. The selected dicarboximides offer new scaffolds for further development as anticancer drugs.

## 1. Introduction

Cancer diseases are an important problem in the clinical medicine and pharmacology of the 21st century. Annually, more than eight million people die from cancer worldwide. In Europe alone, three million new cases are diagnosed and 1.7 million people die from cancer. In Poland, cancer kills nearly 110,000 people, of which about 100,000 die from malignant tumors. It is estimated that the ongoing increase in the incidence of cancer will make it the main cause of death in Poland and worldwide in the coming decades [1,2]. Despite recent progress in cancer treatment (for example development of immune checkpoint inhibitors), chemotherapy (either alone or in combination with radiotherapy or biologic drugs) still remains the first-line treatment in the majority of cancers. Therefore, new cytotoxic low molecular weight compounds are actively sought and developed because they may offer better pharmacokinetic properties and/or less toxic profiles compared to already applied chemotherapeutics (for example platinum compounds or taxanes).

**Figure 1:**
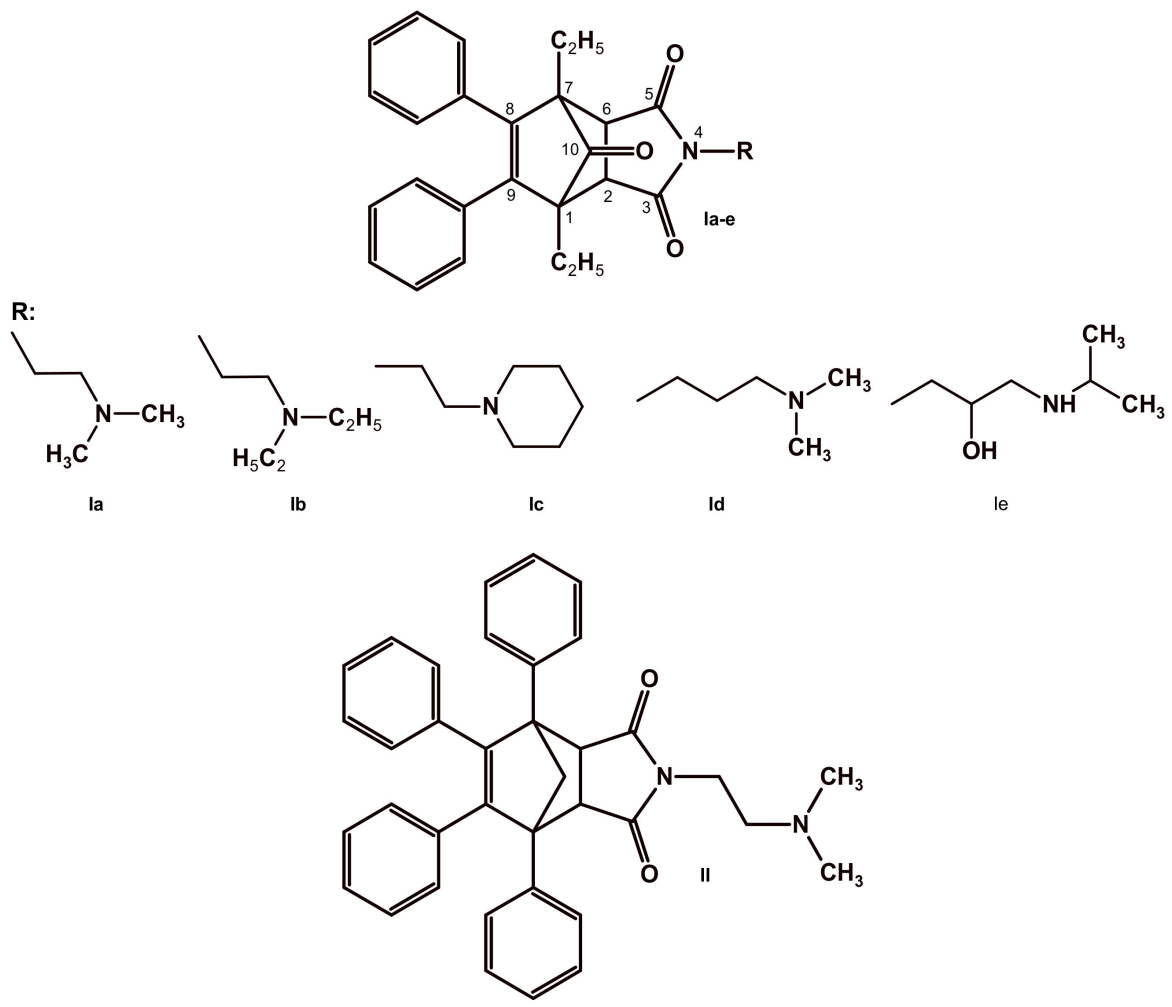
Structures of lead compounds **Ia**–**e** and **II**.

Dicarboximides comprise a large group of compounds and can be divided into four categories: succinimides, maleimides, glutarimides, and phthalimides. Succinimides and phthalimides offer a wide range of chemical and biological applications. They are also very important from a therapeutic perspective, as they are used as drugs (for example, anticonvulsant–ethosuximide) or tested as drug candidates. These derivatives show antifungal, antibacterial, antidepressant, or analgesic activities. Several reports describe the antitumor activity of succinimides and phthalimides. Pyridinyl- and quinolinyl-derivatives of succinimide demonstrated antitumor activity in leukemia cells by inhibition of DNA synthesis [3]. Trifluoromethylated mono- and bicyclic succinimides were found to inhibit the growth of myeloma, non-small cell lung cancer, and renal cancer cells [4]. N-benzylcantharidinamide promoted the degradation of matrix metalloproteinase-9 (MMP-9) mRNA in an antigen R-dependent manner in human hepatocellular carcinoma Hep3B cells. This resulted in decreased expression of MMP-9 and reduced invasion of the highly metastatic Hep3B cells [5]. Benzothiazole derivatives of phthalimide showed significant cytotoxicity and induced apoptosis in human hepatoma (SKHep1), Burkitt’s B-cell lymphoma (CA46), and chronic myelogenous leukemia (K562) cells [6]. Dual-acting thiazolidinone derivates of phthalimide with antiproliferative and immunosuppressive activity were also identified. These compounds inhibited the proliferation of cancer cells and blocked the secretion of pro-inflammatory cytokines, for example, TNF-α, IL-2, or Il-6 [7]. Probably the best-known phthalimide-based drug is thalidomide (N-α-phthalimidoglutarimide). This is a synthetic derivative of phthalimide N-substituted with glutarimide. Since 1957, thalidomide has been used as an anti-emetic agent, but in 1961 it was withdrawn from the market because of severe teratogenicity. However, further studies demonstrated the potent anti-angiogenic and anti-inflammatory activity of thalidomide (and its derivatives—lenalidomide and pomalidomide), which warranted its return as an essential medicine. Currently, thalidomide is used as an anti-cancer drug (for multiple myeloma), for the treatment of myelodysplastic syndrome and inflammatory disorders, like erythema nodosum leprosum [8]. We have previously identified several lead dicarboximides (in particular **Ic**, **Ie**, and **II**, Figure 1) structurally related to succinimides and phthalimides, which exhibited high cytotoxicity and selectivity toward leukemia cells.

We also found that they targeted the ABC50 protein, showed immunomodulatory activity, and induced apoptosis in leukemia cells [9,10,11]. In particular, compound **Ic** inhibited endonuclease-catalyzed digestion of DNA, suggesting its interaction with nucleic acids [9] and activated caspase 8 and 9, which are markers of receptor-mediated and mitochondrial apoptotic pathways, respectively [11]. However, the detailed mechanism of apoptosis and the involvement of mitogen-activated protein kinases (MAPKs) were not investigated. These anticancer and immunomodulatory activities of dicarboximides indicate their importance as potential drug candidates and significance for further development.

The main goal of this study was to obtain new derivatives of dicarboximides with decreased lipophilicity and improved anticancer activity as compared to the lead compounds (i.e., **Ia**–**Ie** and **II**). To achieve this aim, we designed, synthesized, and examined the biological activity of new derivatives of dicarboximides. These new derivatives showed potent cytotoxic and proapoptotic activity in cancer cells, and DNA intercalating properties. Microarray analysis of gene expression revealed that dicarboximides upregulated genes involved in receptor-mediated and mitochondrial apoptotic pathways. Interestingly, antiapoptotic genes associated with NF-kB signaling were also upregulated. They also activated stress-induced MAPK signaling pathways in cancer cells.

## 2. Results

### 2.1. Synthesis

**Scheme 1:**
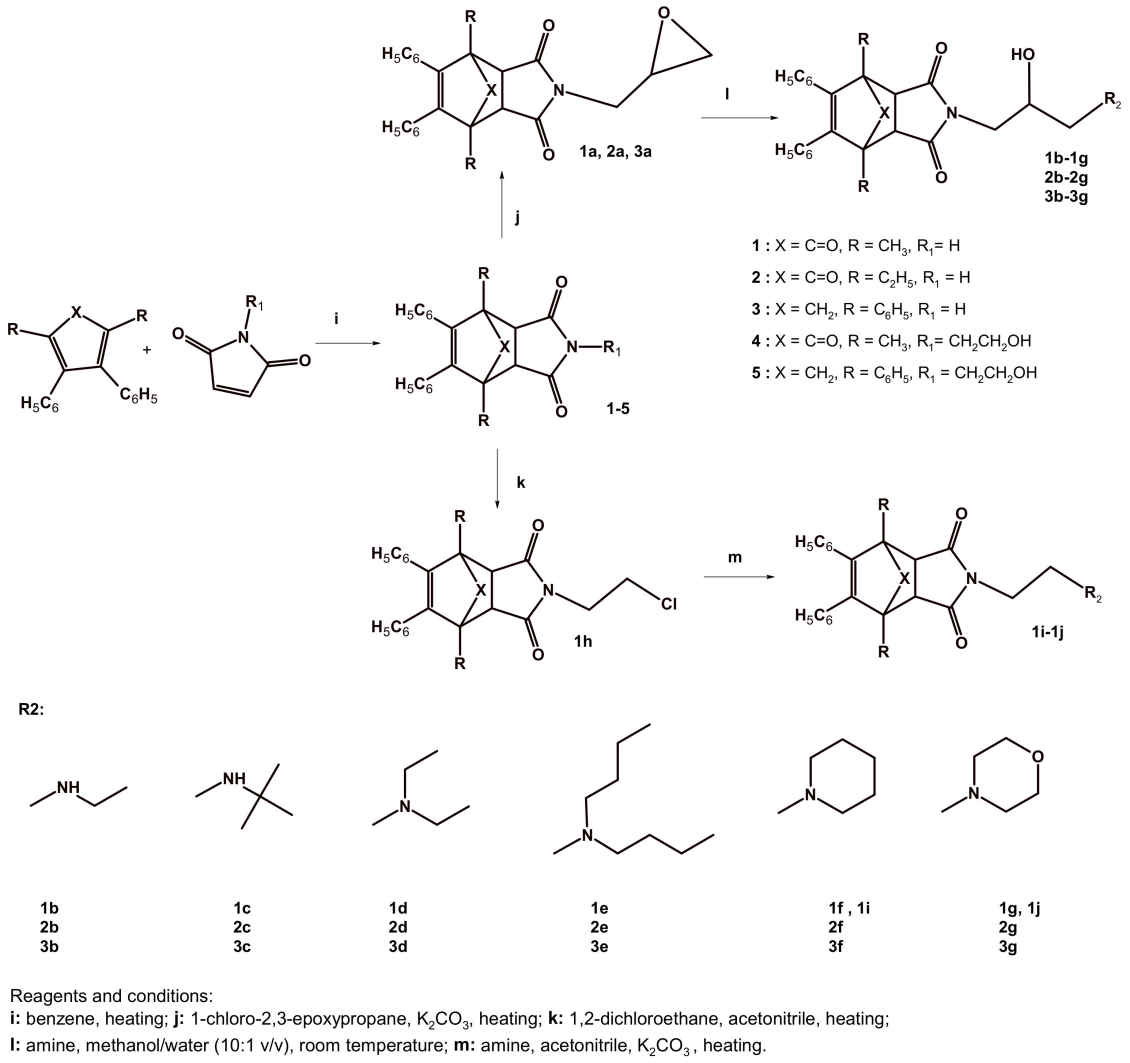
Synthesis of starting imides, N–alkanolamine and N–alkylamino derivatives of dicarboximides.

The starting imides **1**–**3** were obtained in Diels–Alder reactions between appropriate dienes and maleimide, while the compounds **4** and **5** were obtained in the reaction with N-(hydroxyethyl)-maleimide (Scheme 1). The epoxy-derivatives were obtained by condensation of starting imides with epichlorohydrin, and the products were condensed with appropriate amines to give alkanolamine derivatives (Scheme 1). In the reaction between imide **1** and 1,2-dichloroethane, the chloroalkyl derivative was obtained and condensed with an appropriate amine (Scheme 1). All obtained amino-derivatives were converted to their hydrochlorides by using methanol saturated with hydrochloride. The lead compounds with the best activity (**Ic**, **Ie**, and **II**) described previously [9] were converted to their acetates by using boiling acetic acid. The structures of new compounds were established by spectroscopic methods (1H NMR, 13C NMR, and HRMS). In this case, ethyl groups in positions 1 and 7 were replaced by methyl groups.

The main goal of the synthetic part was the synthesis of new derivatives of dicarboximides with decreased lipophilicity in comparison to the lead compounds, i.e., **Ia**–**Ie** and **II**. To reduce the lipophilicity of the new dicarboximides, a hydroxyalkyl chain connected with an aliphatic amine was introduced to the imides (Scheme 1). At the same time, the imide scaffold in derivatives from group **2** and **3** was the same as in lead compounds **Ia**–**Ie** and **II**, respectively (Figure 1). The structure of the imide was changed in derivatives **1b**–**1g**. The lipophilicity of new derivatives was calculated using the Osiris Property Explorer program. For some compounds, lipophilicity was determined experimentally by a reversed-phase chromatography (detailed description is given in Materials and Methods and in the Supplementary Data, Table S2). Table 1 summarizes the calculated (*clogP*) and experimental (*logk_w_*) parameters of the lipophilicity of new derivatives.

In some cases, the new derivatives had lower lipophilicity in comparison to the lead compounds. For example, derivatives of imide **1** had a median *clogP* and *logk_w_* of 3.19 and 4.85, respectively, while corresponding values for lead dicarboximides **Ia**–**Ie** were 4.27 and 5.05. This decrease in lipophilicity can be attributed to the replacement of ethyl groups by methyl groups in the imide scaffold and the presence of the hydroxyalkyl chain. The introduction of a hydroxyalkyl chain in derivatives from the group of imide **2** resulted in a slight decrease in lipophilicity (median *clogP* and *logk_w_* of 4.1 and 5.03, respectively) compared to parent compounds **Ia**–**Ie**. However, pronounced exceptions were alkanoldibutylamine derivatives **1e** and **2e**, which showed significantly increased lipophilicity. The modifications introduced to imide **3** derivatives did not significantly affect their lipophilicity (median *clogP* and *logk_w_* of 5.69 and 6.28, respectively) compared to the lead dicarboximide **II** (*clogP* 5.45 and *logk_w_* 6.34).

### 2.2. MTT Cytotoxicity Studies

The cytotoxicity of new derivatives of dicarboximides was tested in HeLa (cervix carcinoma cell line), K562 (myelogenous leukemia cell line), MOLT-4 (Human T-Lymphoid Cell Line), and HUVEC (normal endothelial) cells. First, the viability of cells was determined after 48 h incubation with the screened compounds used at the concentration of 100 μM (data not shown). For compounds that reduced the viability of cancer cells by more than 50%, the IC_50_ values were determined (Table 2). Cells exposed to 1% DMSO (as vehicle) served as the control with 100% survival. Cells treated with 1 µM staurosporine served as the internal control of the cytotoxicity experiments (the viability of cancer cells was reduced to 44–49%, data not shown).

Test compounds showed significant cytotoxicity toward all tested cancer cell lines. Derivatives of imide **2** and **3** (i.e., Ic*CH_3_COOH, Ie*CH_3_COOH, **2b**–**2g**, **3c**–**3g**, **5**), possessing the same imide skeleton as the lead compounds, displayed the best activity with IC_50_ in the range of 1.9–26 μM. In the group of derivatives of imide **1**, the highest activity was demonstrated by **1e** against all tested cancer cell lines (IC_50_ of 3.2, 5.8, and 8 μM, respectively). It is noteworthy that derivatives **1b**, **1h**, and **1i** were the most toxic to MOLT-4 cells (IC_50_ of 7, 20, and 15 µM, respectively), whereas compound **1f** was cytotoxic to K562 (IC_50_ of 18 µM). Other derivatives of this class showed moderate activity (IC_50_ in the range of 28–105 µM). Similar cytotoxicity as for cancer cells was observed in normal endothelial cells, with IC_50_ values in the range of 0.3–64 µM.

### 2.3. Activation of Apoptosis and Expression Profile of Apoptotic Genes

**Figure 2:**
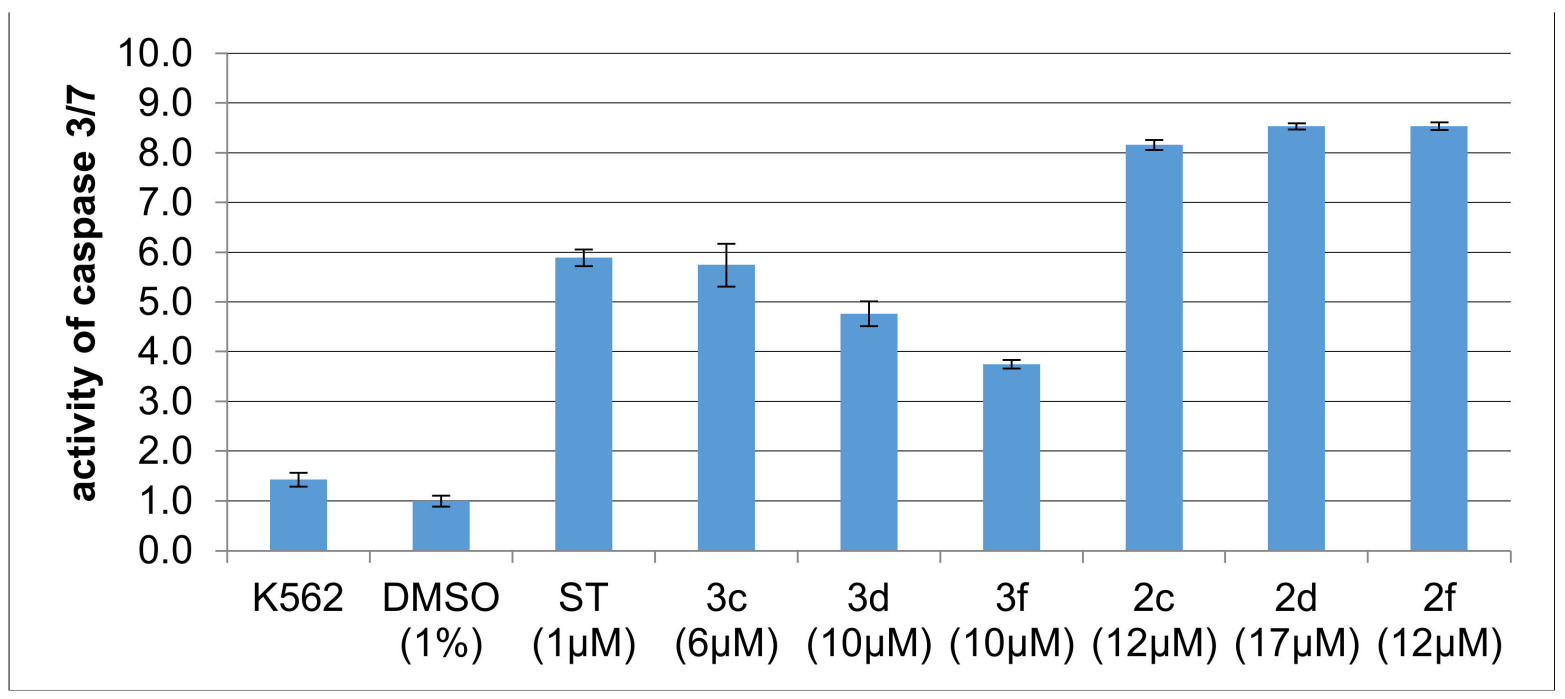
Activity of caspase 3/7 in K562 cells treated with the test dicarboximides or staurosporine for 18 h. Abbreviations: K562—untreated cells; DMSO—K562 cells treated with 1% DMSO; ST—staurosporine. The activity of caspase 3/7 in cells exposed to 1% DMSO was normalized to 1.0. Mean values ± SD are shown.

Six derivatives of dicarboximides, i.e., **3c**, **3d**, **3f**, **2c**, **2d**, and **2f**, with the highest cytotoxicity against K562 leukemia cells (IC_50_ < 6 µM) and with the same alkanolamine fragment in the structure were further evaluated for the ability to induce apoptosis in cancer cells. Caspases 3 and 7 (caspase 3/7) are markers of apoptosis, and their activity was upregulated during this process. K562 cells were treated with 1% DMSO (negative control), 1 μM staurosporine (a strong inducer of apoptosis, a positive control), or a test compound at the concentration of 5xIC_50_. After incubation for 18 h, K562 cells were lysed and the activity of caspase 3/7 was measured using a profluorescent peptide substrate. Staurosporine strongly activated caspase 3/7 in K562 cells, while DMSO had no effect (Figure 2). We also observed a significant increase in the activity of caspase 3/7 in the presence of all tested dicarboximides. Particularly, derivatives **2c**, **2d**, and **2f** were potent activators of apoptosis, as they induced an over eight-fold increase in caspase activity compared to control. Incubation of cells with compounds **3c**, **3d**, and **3f** activated caspase 3/7 to a lesser extent. Altogether, these data suggest that the cytotoxicity of new derivatives of dicarboximides is most likely due to the induction of apoptosis in the chronic myelogenous leukemia (CML) K562 cells.

**Figure 3:**
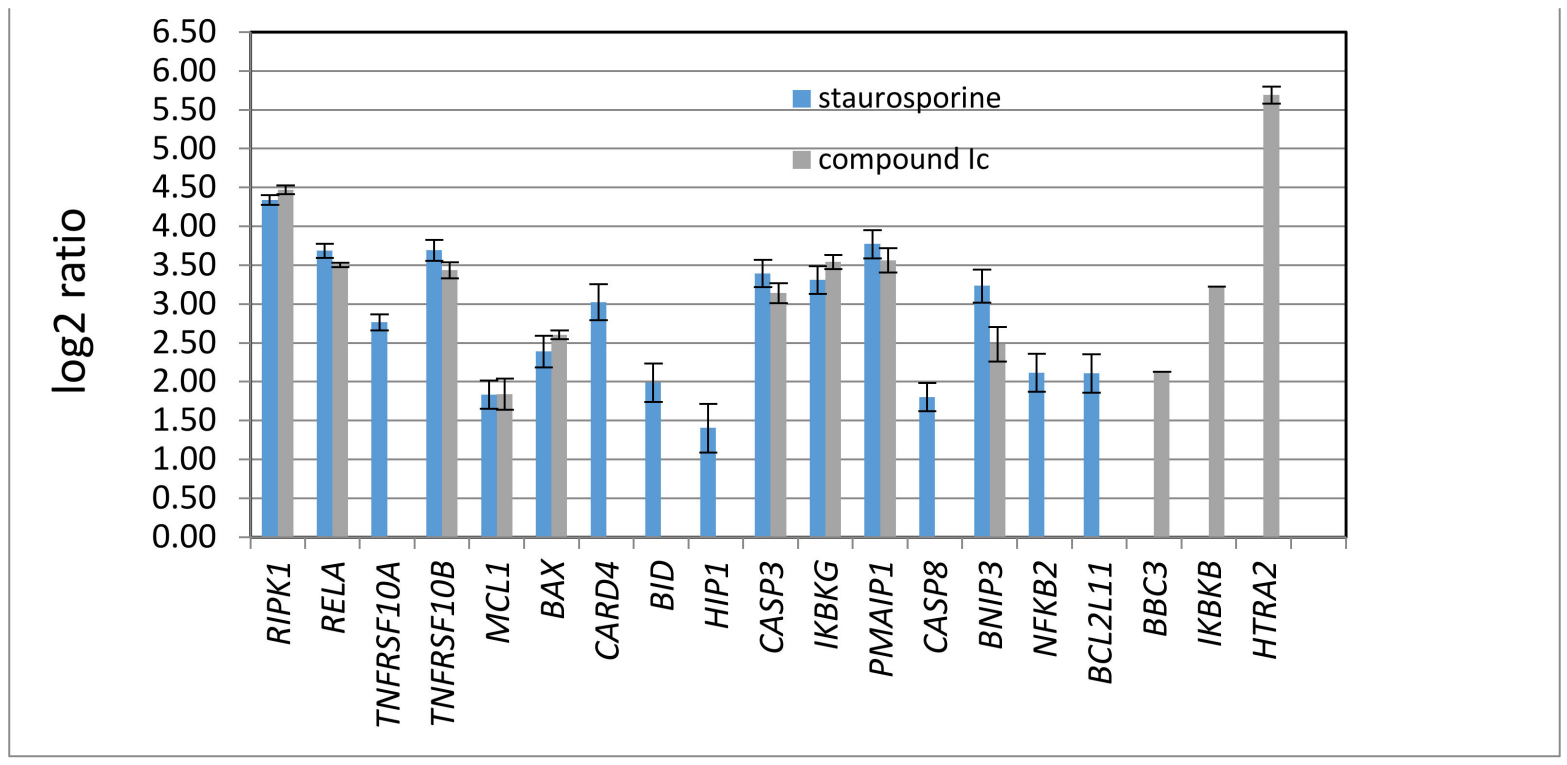
Human apoptosis array analysis of gene expression in K562 exposed to compound **Ic** at 10 µM (5 × IC_50_) for 18 h. Staurosporine (1 µM) served as a positive control. Only apoptosis-related genes with statistically significant changes in expression are shown (*p* < 0.05, *t*-Student). The fold of change was expressed as log2 ratio of gene expression in a test sample vs control sample (cells + DMSO).

We also profiled the expression of 93 genes associated with apoptosis in leukemia cells treated with the lead dicarboximide **Ic**. We showed previously that compound **Ic** induced apoptosis in CML K562 cells and acute lymphoblastic leukemia (ALL) cells MOLT-4, and that both receptor-mediated and mitochondrial apoptotic pathways may be involved [9,11]. K562 leukemia cells were incubated for 18 h with 1% DMSO (control–comparator), staurosporine (1 µM, positive control), or compound **Ic** (10 µM). Subsequently, the total RNA was isolated and the expression of 93 mRNAs associated with apoptosis was determined using DNA microarrays. The expression profile of apoptotic genes upon treatment with **Ic** was similar to staurosporine, a known proapoptotic agent (Figure 3 and Supplementary Materials Table S1). The expression of 12 genes was significantly upregulated in response to **Ic** (Figure 3).

Eight of these genes encode proteins with proapoptotic activity. For example, TNFRSF10B (a membrane receptor from the TNF family) and RIPK1 (a Ser-Thr kinase, which is a key regulator of TNF-mediated apoptosis) activate the receptor pathway of apoptosis. PMAIP1 (Phorbol-12-Myristate-13-Acetate-Induced Protein 1), BAX (BCL2 Associated X, a Bcl-2 family member), BBC3 (BCL2 Binding Component 3, a Bcl-2 family member), and BNIP3 (BCL2 Interacting Protein 3) induce apoptosis via the mitochondrial pathway. HTRA2 (HtrA Serine Peptidase 2) promotes apoptosis by binding and inhibiting IAPs (inhibitor of apoptosis proteins), while CASP3 (caspase 3) is a major executioner protease during cell apoptosis. Only one of the upregulated genes encodes a protein that inhibits apoptosis, i.e., MCL1 (Myeloid Cell Leukemia Sequence 1), which belongs to the Bcl-2 family. Interestingly, compound **Ic** also increased the expression of genes that encode anti-apoptotic proteins involved in the activation of the transcription factor NF-kB. Specifically, IKBKB (Inhibitor Of Nuclear Factor Kappa B Kinase Subunit Beta) and IKBKG (Inhibitor Of Nuclear Factor Kappa B Kinase Subunit Gamma) are protein kinases that activate NF-kB in cells, and RelA is a subunit of NF-kB heterodimer.

### 2.4. Effect of Dicarboximides on MAP Kinase Signaling Pathways

**Figure 4:**
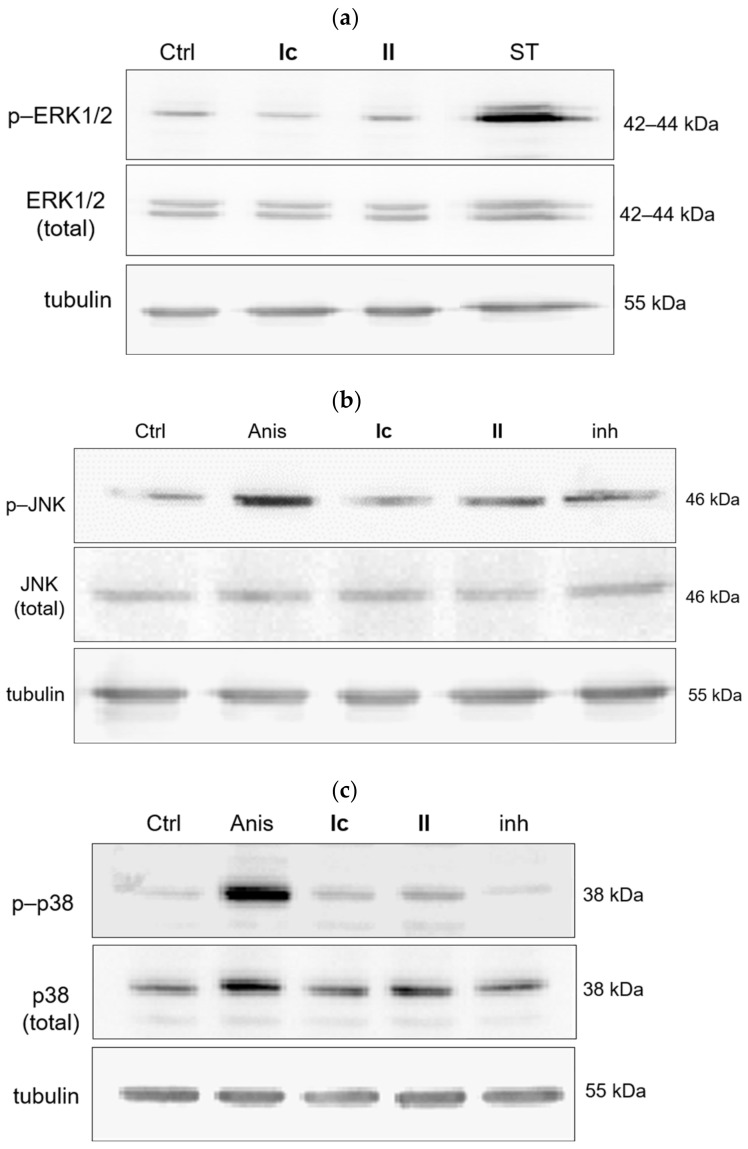
Effect of dicarboximides **Ic** and **II** on activation of ERK1/2 (**a**), JNK (**b**), and p38 (**c**) kinases in K562 leukemia cells. Ctrl—control cells treated with 1% DMSO; Anisomycin 20 µM (positive control, activator of JNK and p38 kinases); **Ic** (6 µM); **II** (3 µM); ST—Staurosporine 1 µM (positive control, activator of ERK1/2); inh—JNK inhibitor 20 µM or p38 inhibitor 10 µM.

In this study, we have demonstrated that new derivatives of dicarboximides are cytotoxic and induce apoptosis in leukemia cells. Similar effects were observed for previously studied lead compounds **Ic** and **II** [9,11]; therefore, we investigated whether mitogen-activated protein kinases (MAPKs) were involved in mediating dicarboximides-induced cytotoxic effects and induction of apoptosis. MAPKs include extracellular signal-regulated kinases (ERK1/2), p38 kinase, and c-Jun N-terminal kinase (JNK), which regulate cellular responses to multiple stimuli like growth factors, cytokines, stress signals, or cytotoxic drugs. In cells exposed to stress conditions (for example toxic compounds), p38 kinase and JNK kinase are often activated, leading to cell cycle arrest and apoptosis. To investigate the effect of dicarboximides on MAPKs, leukemia cells were incubated with lead compounds **Ic** and **II**, and subsequently, cell lysates were immunoblotted with specific antibodies recognizing activated (i.e., phosphorylated) forms of MAP kinases. As positive controls, we used cells treated with staurosporine (activates ERK1/2) or anisomycin (activator of JNK and p38 kinases). As expected, the incubation of K562 cells with compounds **Ic** or **II** had no effect on the ERK pathway, which is usually activated by growth factors and promotes cell proliferation (Figure 4a). However, dicarboximides **Ic** and **II** activated JNK and p38 kinases. This is evidenced by increased phosphorylation of JNK and p38 kinase (Figure 4b,c, lanes 3 and 4) compared to control (lane 1). These data suggest that in leukemia cells dicarboximides activate stress signaling pathways regulated by JNK and p38 kinases, which subsequently leads to apoptotic death.

### 2.5. Interaction with DNA

**Figure 5:**
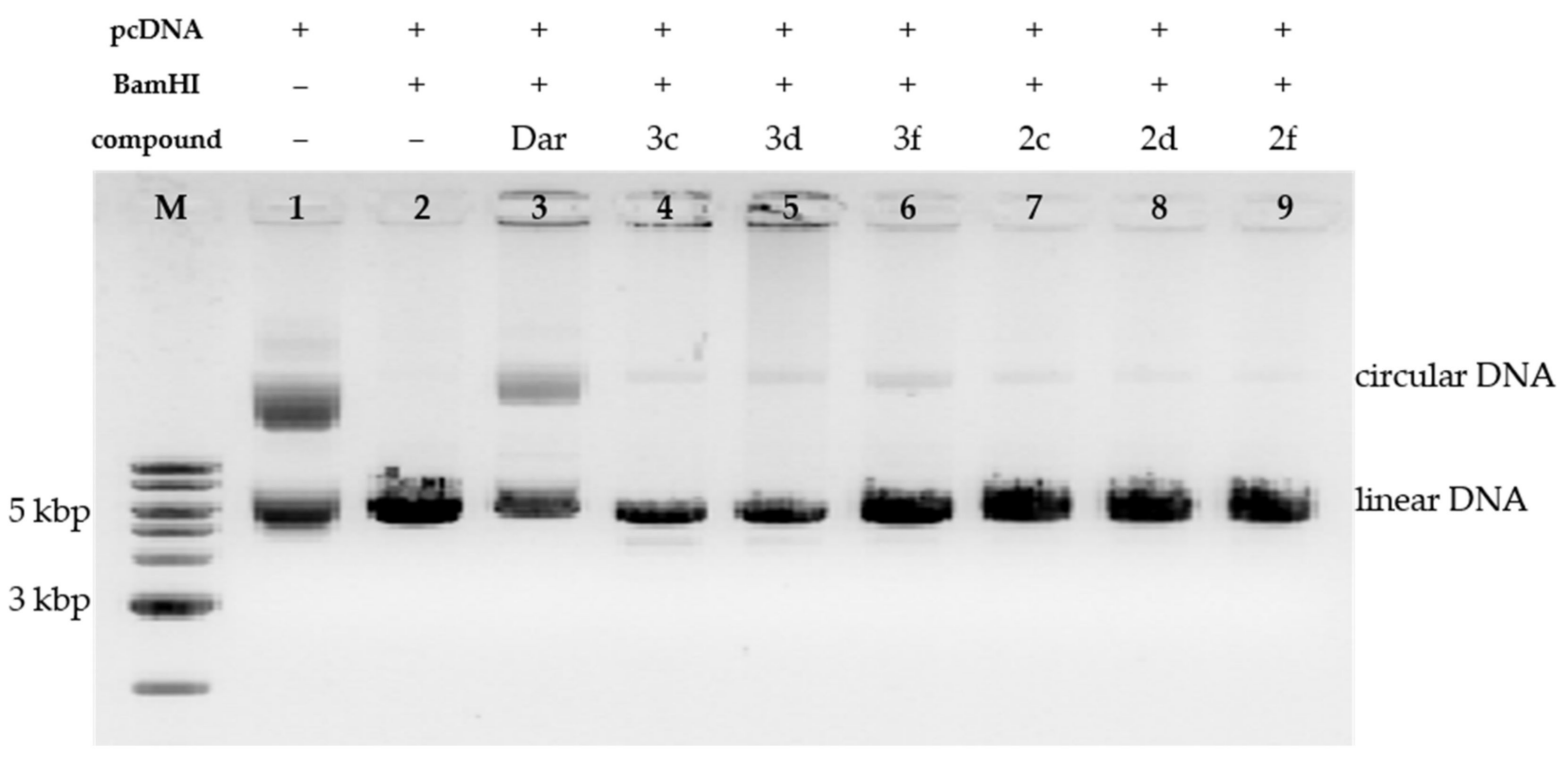
Effect of dicarboximides on digestion of pcDNA3.1HisC (total length 5.5 kbp) with *BamH1* endonuclease observed by agarose gel electrophoresis. M—marker DNA; Dar—daunorubicin.

A DNA-cleavage protection assay has been successfully used for the identification of compounds that bind DNA either covalently or noncovalently. Such binding may result in DNA being less susceptible to enzymatic cleavage [12]. Using this assay, we showed previously that compound **Ic** may interact with DNA [9,10]. It was also reported that naphtalenoimides or indolomaleimides intercalate into DNA [13,14]. With the aid of circular dichroism (CD) spectroscopy, we demonstrated the direct binding of **Ic** to DNA (Supplementary Materials Figure S1). In CD studies, a synthetic 23-nt duplex DNA or bovine thymus DNA was incubated with compound **Ic** or daunorubicin (reference compound). CD spectra indicated that compound **Ic** induced conformational changes in DNA compared to the control sample (Supplementary Materials Figure S1). This observation suggests that the cytotoxicity of dicarboximides may result from binding to and damaging the double-stranded DNA. Using a DNA-cleavage protection assay, we investigated whether selected compounds **2c**, **2d**, and **2f** and **3c**, **3d**, and **3f** interact with a plasmid DNA, resulting in increased resistance to endonucleolytic cleavage. Plasmid DNA (pcDNA3.1HisC) containing a single *BamHI* cleavage site was incubated with test compounds and subsequently digested with *BamH1* restriction endonuclease. Daunorubicin (a strong DNA-intercalating agent) was used as a reference compound. Non-digested plasmid DNA existed predominantly in the circular form; however, the superhelical and linear forms were also detected (Figure 5, lane 1). Digestion with the *BamH1* enzyme converted the plasmid into a linear form (Figure 5, lane 2; no circular DNA), while daunorubicin (at 100 µM) prevented linearization of DNA (Figure 5, lane 3; circular DNA present). In the presence of test dicarboximides, the linear plasmid DNA was predominant; however, the circular form was also detected (Figure 5 lane 4–9). This indicates that test dicarboximides interact with DNA. Interestingly, this interaction seems to be stronger for **3c**, **3d**, and **3f** (as more circular DNA is observed in lanes 4–6) than for the remaining test compounds (lanes 7–9). This observation can be explained by the higher lipophilicity of **3c**, **3d**, and **3f** compared to **2c**, **2d**, and **2f**, which may facilitate their more efficient binding to DNA.

## 3. Discussion

In our previous studies, we identified several derivatives of dicarboximides (**Ia**–**Ie**, **II**) that efficiently killed cancer cells and were not toxic to normal endothelial cells [9,10,11]. Compounds **Ic**, **Ie**, and **II** were particularly promising, as they showed specificity to cancer cells only and induced apoptosis. However, they are lipophilic (Table 1) and have limited water solubility. Based on their structures, we have designed and synthesized a library of 26 new derivatives of dicarboximides with lower lipophilicity, which is a desired property for potential new drug candidates. This was achieved mainly by the replacement of ethyl groups in the imide scaffold by methyl groups at positions 1 and 7 as well as by the introduction of a side chain with the hydroxyalkyl group substituted with alkylamino groups (Scheme 1). This resulted in significantly lower lipophilicity of derivatives of imide **1** (median *clogP* 3.19), and imide **2** (median *clogP* of 4.1) compared to parent compounds **Ia**–**Ie** (median *clogP* 4.27). However, chemical modifications introduced to derivatives of imide **3** rather increased their lipophilicity (median *clogP* 5.69) when compared to the parent dicarboximide **II** (*clogP* 5.45) (Table 1). Lipophilicity is the most important property of new drug candidates. According to Lipinski’s rule, *clogP* > 5 predicts poor absorption and/or permeability of a drug [15]. In addition, high lipophilicity may increase the unspecific cytotoxicity [16].

The cytotoxic properties of all investigated analogs were tested in human cells of cancer and normal origin. Fourteen compounds (**1b**, **1e**, **2b**–**2f**, **3c**–**3g**, **5**, Ie*) displayed significant cytotoxicity toward cancer cell lines (IC_50_ < 10 μM); however, they were less toxic than the lead compounds and exhibited cytotoxicity in normal endothelial cells (Table 2). Comparison of IC_50_ values indicates that compounds with lower lipophilicity are less cytotoxic. This is particularly evident for derivatives of dicarboximid **1**, which show the lowest cytotoxicity.

Preliminary studies on derivatives **3c**, **3d**, **3f**, **2c**, **2d**, and **2f** showed that these compounds activated caspase 3 and 7 in K562 cells (Figure 2). This suggests that the cytotoxicity of the new derivatives is associated with the induction of apoptosis in CML cells. Interestingly, proapoptotic activity of dicarboximides seems to depend on lipophilicity. Derivatives of imide **2** (median *clogP* 4.1) were significantly more potent than derivatives of imide **3** (median *clogP* 5.69). Apoptosis may proceed through two major pathways: receptor-mediated (extrinsic) and mitochondrial (intrinsic) [17]. The effect of dicarboximides on apoptotic pathways was investigated by microarray-based gene expression profiling. We found that several pro-apoptotic genes involved in the receptor and mitochondrial apoptotic pathways were upregulated in K562 cells treated with **Ic**. The expression profile induced by **Ic** was similar to staurosporine, a natural compound that activates mainly the mitochondrial pathway of apoptosis [18]. Specifically, compound **Ic** upregulated the expression of TNFRSF10B and *RIPK1*, which promote apoptosis via the receptor (extrinsic) pathway. TNFRSF10B belongs to a TNF family of transmembrane receptors. Upon binding the TRAIL ligand (TNF-related apoptosis-inducing ligand), TNFRSF10B is activated leading to caspase 8-mediated apoptosis [19]. Interestingly, in response to cytotoxic agents cancer cells overexpress TNFRSF10B, and this seems to be sufficient to trigger ligand-independent apoptosis [20]. RIPK1 (Receptor Interacting Serine/Threonine Kinase 1) is involved in signaling events downstream of TNFR1 that (depending on the cellular context) may lead to apoptosis through recruitment of FADD and subsequent activation of caspase 8 [21] or promote necroptosis through phosphorylation of RIPK3 and MLKL [22]. Compound **Ic** also increased the expression of protein genes involved in the mitochondrial (intrinsic) pathway of apoptosis: *PMAIP1* (Phorbol-12-Myristate-13-Acetate-Induced Protein 1), *BAX* (BCL2 Associated X, a Bcl-2 family member), *BBC3* (BCL2 Binding Component 3, a Bcl-2 family member), and *BNIP3* (BCL2 Interacting Protein 3). BAX is a member of the Bcl-2 family of proteins and a key activator of the intrinsic apoptotic pathway. In response to cellular stress, BAX is activated and oligomerizes, forming pores in the mitochondrial outer membrane. This enables cytochrome c and/or SMAC/Diablo proteins to leak into the cytoplasm and activate caspase 3 [23]. BBC3 is a pro-apoptotic protein that belongs to a class of BH3-only proteins. BBC3 binds other anti-apoptotic Bcl2 proteins (for example antiapoptotic MCL1) using its BH3 domains and thus promotes apoptosis. Interestingly, the expression of BBC3 is induced by DNA damaging agents and regulated by p53 [24]. This could explain the increased expression of BBC3 in K562 cells treated with **Ic**, as we demonstrated previously that this compound interacts with DNA and thus may be considered a DNA-damaging agent [9]. Altogether, these results suggest that dicarboximides activate both receptor-mediated and mitochondrial pathways of apoptosis by the upregulation of several genes involved in both pathways.

Interestingly, compound **Ic** also increased the expression of anti-apoptotic genes encoding IKBKB/IKKβ (Inhibitor Of Nuclear Factor Kappa B Kinase Subunit Beta), IKBKG/IKKγ (Inhibitor Of Nuclear Factor Kappa B Kinase Subunit Gamma), and RelA, which serves as a subunit of NF-κB heterodimer. These proteins are involved in the activation and function of transcription factor NF-κB, which regulates the expression of several genes involved in inflammation, immune responses, cell survival, and tumorigenesis and inhibits apoptosis. Serine kinase IKKβ and regulatory protein Ikkγ are key components of the IKK complex, which is essential for the activation of NF-κB transcription factors. The anti-apoptotic activity of IKKβ and IKKγ has been confirmed in studies using knockout mice. IKKβ-deficient mice died during embryonic development due to the uncontrolled apoptosis of liver cells [25]. A similar phenotype, i.e., liver apoptosis was observed in the case of Ikkγ-deficient male embryos, leading to their death on day (E)12 [26].

The cytotoxic effects of dicarboximides may also be mediated by MAPKs, a family of Ser-Thr kinases that consists of ERK1/2, p38, and JNK kinase. We demonstrated that in cells exposed to lead dicarboximides **Ic** and **II**, p38 and JNK kinases are activated while there was no effect on ERK1/2 (Figure 4). ERK1/2 are activated mainly by growth factors (for example PDGF or EGF) and positively control cell proliferation by promoting G1- to S-phase transition [27]. On the other hand, p38 and JNK kinases are activated by stress conditions, for example, oxidative stress, ionizing radiation, cytotoxic/genotoxic agents, DNA damage, inflammatory cytokines, or deprivation of growth factors. In response to stress conditions, JNK transactivates the c-Jun transcription factor or phosphorylates proteins associated with apoptosis, leading to activation of both mitochondrial and receptor-mediated pathways of apoptosis [28]. Similarly, activation of p38 leads to inhibition of cell cycle progression and induction of apoptosis [29,30]. Our results suggest that p38 and JNK can contribute to the activation of apoptosis induced by **Ic** and **II**.

We also examined whether DNA may be targeted by test derivatives. The obtained results showed that dicarboximides inhibited the digestion of plasmid DNA with *BamHI* endonuclease, albeit to a lower extent than daunorubicin (Figure 5). These data suggest that the test dicarboximides may intercalate to DNA and possibly cause DNA damage. This property of dicarboximides (including compound **Ic**) could explain the increased expression of the BBC3 gene in K562 cells exposed to **Ic**, as discussed above.

The SAR analysis indicated that the volume of substituents at positions 1 and 7 of the imide system is an important factor in determining the cytotoxicity of dicarboximides (Figure 1). This conclusion is supported by comparison of the cytotoxic and apoptotic activities in three sets of compounds: **1c**, **2c**, and **3c**; **1d**, **2d**, and **3d**; or **1f**, **2f**, and **3f**. The derivatives containing ethyl or phenyl groups at positions 1 and 7 showed lower IC_50_ towards HeLa, K456, and MOLT-1 cells than compounds with methyl groups. The type of alkylamine substituent in the side chain is also important for inducing cytotoxicity and apoptosis-signaling pathways. Dicarboximides with alkylamine substituent bearing a protonated nitrogen atom at physiological pH (pKa of amines is > 9.0, [31]) were the most active. For example, *tert*-butyl derivatives (**2c** and **3c**), piperidynyl derivatives (**2f** and **3f**), dibutyl derivatives (**1e**, **2e**, and **3e**), and diethylamine derivative (**3d**) showed the highest cytotoxicity against cancer cells tested. It is worth noting that all of the above-mentioned compounds have values of *clogP* > 4. On the other hand, compounds containing the morpholine system i.e., **1g**, **2g**, and **3g** (with the lowest *clogP* values among other derivatives within the corresponding group of imides **1**, **2**, and **3**, respectively) exhibited the lowest cytotoxicity towards tested cell lines. This result suggests that the presence of an acceptor oxygen atom at the morpholine ring, but not the cyclic structure of the substituent, is responsible for undesirable interactions that are not offered by the piperidine ring of **1f**, **2f**, and **3f**. The favorable role of the protonated nitrogen atom of the alkylamino group is further supported by comparison of compounds of scaffold **1**, which are less or not cytotoxic when the imide function is substituted with hydroxyalkyl (**4**) or chloroethyl substituent (**1h**), respectively, but exhibit higher cytotoxicity when **1h** is substituted with a *tert*-butyl amine residue (**1i**). However, the presence of the protonated alkylamino group is not an obligatory feature to provide high cytotoxicity, since, e.g., compound **5**, containing a hydroxyalkyl side chain, exhibited significant cytotoxicity. The activity of the lead compounds is also influenced by the type of salts, i.e., acetate vs. hydrochloride. The acetate salts showed lower cytotoxicity and lacked selectivity against the studied cell lines. It can be hypothesized that the change of polarization of the molecule by the acetate anion and the decrease in the hydrophilicity could affect the activity of these derivatives.

## 4. Materials and Methods

### 4.1. Chemistry

All chemicals used in the reaction were supplied from Sigma-Aldrich (Saint Louis, MO, USA) or POCH (Polskie Odczynniki Chemiczne, Gliwice, Poland). Melting points were determined by the capillary method using the Electrothermal 9100 apparatus and were uncorrected. The nuclear magnetic resonance spectra were recorded in DMSO-d_6_ or CDCl_3_ on VMNRS300 spectrometer (Bruker, Billerica MA, USA) operating at 300 MHz (^1^HNMR) and 75 MHz (^13^CNMR). Chemical shifts (δ) are expressed in parts per million relative to tetramethylsilane (TMS) used as the internal reference. Coupling constants (*J*) values are given in hertz (Hz), and spin multiples are given as s (singlet), d (doublet), t (triplet), and m (multiplet). Mass spectral ESI (electrospray ionization) measurements were carried out on a MicrOTOF II Bruker instrument with a TOF detector. The spectra were obtained in the positive ion mode. Flash chromatography was performed on Merck Kieselgel (Darmstadt, Germany) 0.05–0.2 mm reinst (70–325 mesh ASTM) silica gel using chloroform or chloroform–methanol as an eluent system at the *v*/*v* ratio of 50:0.2 or 50:0.5. The progress of the reactions described in the experimental section was monitored by TLC on silica gel (plates with fluorescent indicator 254 nm, layer thickness 0.2 mm, Kieselgel G. Merck), using chloroform–methanol as an eluent system at the *v*/*v* ratio of 9.8:0.2 or 9.5:05.

#### 4.1.1. Synthesis of Imides **1**–**5** and **1h**

The starting imides **1**–**3** were prepared by the methods described earlier [10,32].

#### 4.1.2. Synthesis of 1,7-Dimethyl-8,9-diphenyl-4-azatricyclo[5.2.1.0^2,6^]dec-8-ene-3,5,10-trione (**1**)

A mixture of 2,5-dimethyl-3,4-diphenylcyclopenta-2,4-dien-1-one (0.02 mol) and maleimide (0.022 mol) was heated for 6 h in 20 mL of benzene. During the synthesis, the solution became red, and a white solid precipitated from it. When the reaction was completed, the solvent was evaporated under reduced pressure, and the solid residue was crystallized from benzene.

M.W. = 357.4018; C_23_H_19_NO_3_; Yield: 77%, white powder, m.p. 220.6–221.1 °C (from benzen); ^1^HNMR (300 MHz, DMSO-d_6_+ TMS, δ/ppm): 1.39 (6H, *s*, -CH_3_); 3.38 (2H, *s*, C2-H, C6-H); 6.98 (4H, *m*, Ar-H); 7.19 (6H, *m*, Ar-H); 11.66 (1H, *s*, NH); Anal. Calc for C_23_H_19_N_3_O_3_: 77.28% C; 5.32% H, 3.92% N; found 77.40% C; 5.30% H; 3.99% N; ^13^CNMR: δ 11.7, 48.5, 55.5, 127.4, 127.9, 129.4, 133.2, 141.3, 177.5, 199.5; HRMS (*m*/*z*): calculated value for [M + Na^+^] 380.1257; found 380.1259.

#### 4.1.3. Synthesis of 1,7-Diethyl-8,9-tetraphenyl-4-azatricyclo[5.2.1.0^2,6^]dec-8-ene-3,5,10-trione (**2**)

A mixture of 2,5-diethyl-3,4-diphenylcyclopenta-2,4-dien-1-one (0.01 mol) and maleimide (0.012 mol) in benzene (15 mL) was refluxed for 14 h. When the reaction was completed, the solvent was evaporated under reduced pressure, and the solid residue was crystallized from benzene.

M.W. = 385.455; C_25_H_23_NO_3_; Yield: 92%; white powder, m.p. 206.8–209 °C (from benzen); ^1^HNMR (300 MHz, DMSO-d_6_+ TMS, δ/ppm): 0.87 (6H, *t*, *J* = 7.5 Hz, -CH_3_); 2.05 (m, 4H, -CH_2_-), 3.58 (2H, *s*, C2-H, C6-H); 6.97 (4H, *m*, Ar-H); 7.18 (6H, *m*, Ar-H); 11.70 (1H, *s*, NH); Anal. Calc for C_25_H_23_NO_3_: 77.93% C; 5.98% H, 3.63% N; found 77.94% C; 5.97% H; 3.62% N; ^13^CNMR: δ 9.1, 19.2, 43.9, 55.3, 60.4, 128.0, 129.4, 133.1, 142.4, 171.6, 197.8; HRMS (*m*/*z*): calculated value for [M + Na^+^] 408.1570; found 408.1570.

#### 4.1.4. Synthesis of 1,7,8,9-Tetraphenyl-4-azatricyclo[5.2.1.0^2,6^]dec-8-ene-3,5-dione (**3**)

A mixture of 1,2,3,4-tetraphenylcyclopenta-1,3-diene (0.02 mol) and maleimide (0.022 mol) was heated for 6 h in 20 mL of benzene. When the reaction was completed, the solvent was evaporated under reduced pressure, and the solid residue was crystallized from benzene.

M.W. = 467.5571; C_33_H_25_NO_2_; Yield: 81%, white powder, m.p. 246.5–246.9 °C (from benzen); ^1^HNMR (300 MHz, DMSO-d_6_+ TMS, δ/ppm): 2.23 (1H, *d*, *J* = 8.4, C10-H), 3.23 (1H, *d*, *J* = 8.7, C10-H), 4.20 (2H, *s*, C2-H, C6-H); 6.60 (4H, *m*, Ar-H); 6.88 (6H, *m*, Ar-H); 7.24 (6H, *m*, Ar-H); 7.74 (4H, *d*, *J* = 7.2, Ar-H); 11.44 (1H, *s*, NH); Anal. Calc for C_33_H_25_NO_2_: 84.79% C; 5.35% H, 3.00% N; found 84.85% C; 5.39% H; 3.08% N; ^13^CNMR: δ 52.9, 62.9, 65.1, 126.4, 126.6, 127.1, 127.7, 129.1, 129.6, 134.5, 139.8, 145.0, 178.0; HRMS (*m*/*z*): calculated value for [M + Na^+^] 490.1778; found 490.1777.

#### 4.1.5. Synthesis of N-Hydroxyethylimides **4** and **5**

A mixture of an appropriate diene:

- 2,5-dimethyl-3,4-diphenylcyclopenta-2,4-dien-1-one (0.02 mol) for compound **4**
- 1,2,3,4-tetraphenylcyclopenta-1,3-diene (0.02 mol) for compound **5** and N-hydroxymaleimide (0.022 mol) was heated for 6 h in 20 mL of benzene. When the reaction was completed, the solvent was evaporated under reduced pressure, and the solid residue was crystallized from benzene.

***4-(2-hydroxyethyl)-1,7-dimethyl-8,9-diphenyl-4-azatricyclo[5.2.1.0^2,6^]dec-8-ene-3,5,10-trione (*4*)***

M.W. = 401.4544; C_25_H_23_NO_4_; Yield: 84%; white powder, m.p. 186–187 °C (from MeOH/(Et)_2_O); ^1^HNMR (300 MHz, DMSO-d_6_+ TMS, δ/ppm): 1.57 (6H, *s*, -CH_3_), 2.16 (1H, *s*, -OH), 3.25 (2H, *m*, C2-H, C6-H), 3.73 (4H, *m*, -CH_2_-), 6.93 (4H, *m*, Ar-H), 7.26 (6H, *m*, Ar-H); ^13^CNMR: δ 12.2, 47.4, 47.9, 56.5, 60.1, 127.7, 128.1, 129.4, 132.9, 141.5, 175.8, 176.1, 199.3; HRMS (*m*/*z*): calculated value for [M + Na^+^] 424.1519; found 424.1519.

***4-(2-hydroxyethyl)-1,7,8,9-tetraphenyl-4-azatricyclo[5.2.1.02,6]dec-8-ene-3,5-dione (*5*)***

M.W. = 511.6096; C_35_H_29_NO; Yield: 61%, white powder, m.p. 197–198 °C (from benzen); ^1^HNMR (300 MHz, DMSO-d_6_+ TMS, δ/ppm): 2.12 (1H, *br.s*, -OH), 2.32 (1H, *d*, *J* = 4.5, C6-H), 3.17 (1H, *d*, *J* = 4.5, C2-H), 3.74 (2H, *m*, C1′-H, C2′-H); 4.17 (2H, *s*, C10-H), 6.55 (4H, *m*, Ar-H); 6.89 (6H, *m*, Ar-H); 7.32 (6H, *m*, Ar-H); 7.70 (4H, *m*, Ar-H); ^13^CNMR: δ 42.2, 52.2, 60.5, 63.6, 65.9, 126.8, 127.1, 127.4, 128.1, 129.0, 129.8, 133.8, 139.2, 145.0, 177.1; HRMS (*m*/*z*): calculated value for [M + Na^+^] 543.5988; found 534.2045.

#### 4.1.6. Synthesis of 4-(2-Chloroethyl)-1,7-dimethyl-8,9-diphenyl-4-azatricyclo[5.2.1.0^2,6^]dec-8-ene-3,5,10-trione (**1h**)

To the solution of imide **a** (0.01 mol) in acetonitrile (30 mL), 1,2-dichloroethane (0.02 mol) was added. The reaction was heated for 72 h. When the reaction was completed, the solvent was evaporated under reduced pressure, and the residue was purified by column chromatography on silica gel (eluent: chloroform).

***4-(2-chloroethyl)-1,7-dimethyl-8,9-diphenyl-4-azatricyclo[5.2.1.0^2,6^]dec-8-ene-3,5,10-trione (*1h*)***

M.W. = 419.9000; C_25_H_22_ClNO_3_; Yield: 74%; white powder, m.p. 190–191 °C (from hexane); ^1^HNMR (300 MHz, CDCl_3_, δ/ppm): 1.58 (6H, -CH_3_), 3.28 (2H, *m*, C2-H, C6-H), 3.64 (2H, *t*, *J* = 6.4 Hz, C1′-H,), 3.86 (2H, *t*, *J* = 6 Hz, C2′-H), 6.91 (4H, *m*, Ar-H), 7.25 (6H, *m*, Ar-H); ^13^CNMR: δ 12.2, 39.5, 40.4, 47.9, 56.5, 127.7, 128,1, 129.4, 132.9, 141.5, 175.1, 199.2; HRMS (*m*/*z*): calculated value for [M + Na^+^] 442.8893; found 442.1175.

#### 4.1.7. General Conditions of Synthesis of New N-Alkylamino Derivatives of 1,7-Dimethyl-8,9-diphenyl-4-azatricyclo[5.2.1.0^2,6^]dec-8-ene-3,5,10-trione (**1i**–**1j**)

The 4-(2-chloroethyl)-1,7-dimethyl-8,9-diphenyl-4-azatricyclo[5.2.1.02,6]dec-8-ene-3,5,10-trione was dissolved in acetonitrile (30 mL), and then anhydrous K_2_CO_3_ (0.01 mol) and an appropriate amine (0.01 mol) were added. The reaction was carried out in reflux, respectively, for 24–48 h in the boiling temperature of the solvent. The reaction was monitored by TLC. When the reaction was completed, the solvent was evaporated, and the residue was purified by column chromatography on silica gel (eluent: chloroform or chloroform/methanol 50:0.2 *v*/*v*).

***4-[2-(morpholin-4-yl)ethy]-1,7-dimethyl-8,9-diphenyl-4-azatricyclo[5.2.1.0^2,6^]dec-8-ene-3,5,10-trione (*1j*)***

M.W. = 470.5595 (free amine); C_29_H_30_N_2_O_4_*HCl; Yield: 70%; white powder, m.p. 227–228 °C (from EtOH/(Et)_2_O); ^1^HNMR (300 MHz, DMSO-d_6_+ TMS, δ/ppm): 1.41 (6H, *s*, -CH_3_), 3.09 (2H, *m*, -CH_2_-morph), 3.30 (2H, *m*, -CH_2_-morph), 3.39 (3H, *m*, C2-H, C6-H), 3.86 (4H, *m*, C1′-H, -CH_2_-morph), 3.91 (2H, *m*, C2′-H), 6.87 (4H, *m*, Ar-H), 7.18 (6H, *m*, Ar-H), 11.57 (1H, *br.s*, NH^+^); ^13^CNMR: δ 11.8, 32.5, 48.1, 50.9, 52.3, 55.6, 62.8, 127.5, 128,1, 129.2, 133.1, 141.4, 176.3, 199.2; HRMS (*m*/*z*): calculated value for [M + H^+^] 471.2278; found 471.2276.

***4-[2-(piperidin-1-yl)ethy]-1,7-dimethyl-8,9-diphenyl-4-azatricyclo[5.2.1.0^2,6^]dec-8-ene-3,5,10-trione (*1i*)***

M.W. = 468.5866 (free amine); C_30_H_32_N_2_O_3_*HCl; Yield: 64%; white powder, m.p. 214–216 °C (from EtOH/(Et)_2_O); ^1^HNMR (300 MHz, CDCl_3_, δ/ppm): 1.42 (6H, -CH_3_), 1.76 (4H, *m*, -CH_2_-piper), 2.85 (2H, *m*, C1′-H), 3.16 (2H, *m*, C2′-H), 3.36 (1H, *m*, C6-H), 3.49 (1H, *m*, C2-H), 3.54 (4H, *m*, -CH_2_-piper), 3.82 (2H, *m*, -CH_2_-piper), 6.86 (4H, *m*, Ar-H), 7.19 (6H, *m*, Ar-H), 10.37 (1H, *br.s*, NH^+^); ^13^CNMR: δ 11.8, 15.1, 21.2, 22.0, 32.9, 48.0, 51.9, 55.6, 127.5, 128,1, 129.2, 133.0, 141.4, 176.1, 199.1; HRMS (*m*/*z*): calculated value for [M + H^+^] 469.2486; found 469.2485.

#### 4.1.8. General Conditions of the Synthesis of New Alkanoloamine Derivatives of Dicarboximides (**1b**–**1g**, **2b**–**2g**, **3b**–**3g**)

The new alkanolamine derivatives were obtained in two-step reactions according to the method described previously [10]. Step **I**: the mixture of an appropriate imide:

**1**: 1,7-dimethyl-8,9-diphenyl-4-azatricyclo[5.2.1.0^2,6^]dec-8-ene-3,5,10-trione
**2**: 1,7-diethyl-8,9-diphenyl-4-azatricyclo[5.2.1.0^2,6^]dec-8-ene-3,5,10-trione
**3**: 1,7,8,9-tetraphenyl-4-azatricyclo[5.2.1.0^2,6^]dec-8-ene-3,5-dione

(0.01 mol) with 1-chloro-2,3-epoxypropane (50–60 mL), in the presence of an anhydrous K_2_CO_3_ (0.01 mol) was stirred on a magnetic stirrer under reflux with a tube with CaCl_2_. The reaction was carried out. at room temperature for 15 h. When the reaction was completed (TLC control), the inorganic precipitate was filtered off and the filtrate was concentrated. The obtained oily product was purified by column chromatography on silica gel (eluent: chloroform and chloroform/methanol 50:0.2). Step **II**: to the solution of an appropriate 4-(oxirane-2-ylmethyl)-4-imide (**1a**, **2a**, **3a**) (0.001 mol) in a mixture of methanol/water (10:1 *v*/*v*), the appropriate amine (0.001 mol) was added. Reactions were carried out at room temperature on a magnetic stirrer under reflux for 15–20 h. When the reaction was completed (TLC control), the excess of the solvent was evaporated and the crude product was purified by column chromatography on silica gel (eluent: chloroform or chloroform/methanol (50:0.2, 50:0.5)).

***4-[3-(ethylamino)-2-hydroxypropyl]-1,7-dimethyl-8,9-diphenyl-4-azatricyclo [5.2.1.0^2,6^]dec-8-ene-3,5,10-trione (*1b*)***

M.W. = 458.5488 (free amine); C_28_H_30_N_2_O_4_*HCl; Yield: 55%; white powder, m.p. 198–201 °C (from MeOH/(Et)_2_O); ^1^HNMR (300 MHz, DMSO-d_6_+ TMS, δ/ppm): 1.18 (3H, *t*, *J* = 7.2 Hz, -CH_3_), 1.42 (6H, *s*, -CH_3_), 2.78 (1H, *m*, C6-H), 2.80 (2H, *m*, -CH_2_-) 3.05 (1H, *m*, C2-H), 3.37(2H, *m*, C3′-H), 3.51 (2H, *m*, C1′-H), 4.10 (1H, *s*, C2′-H), 5.88 (1H, *s*, -OH), 6.87 (4H, *m*, Ar-H), 7.18 (6H, *m*, Ar-H), 8.65 (1H, *br.s*, -NH_2_^+^), 9.09 (1H, *br.s*, -NH_2_^+^); ^13^CNMR: δ 10.7, 11.8, 42.3, 42.7, 47.4, 47.6, 49.3, 55.7, 63.4, 127.5, 128,1, 129.3, 133.2, 141.3, 175.8, 176.2, 199.4; HRMS (*m*/*z*): calculated value for [M + H^+^] 459.2284; found 459.2284.

***4-[3-(tert-butylamino)-2-hydroxypropyl]-1,7-dimethyl-8,9-diphenyl-4-azatricyclo [5.2.1.0^2,6^]dec-8-ene-3,5,10-trione (*1c*)***

M.W. = 486.6019 (free amine); C_30_H_34_N_2_O_4_*HCl; Yield: 57%; white powder, m.p. 248–250 °C (from MeOH/(Et)_2_O); ^1^HNMR (300 MHz, DMSO-d_6_+ TMS, δ/ppm): 1.26 (9H, *s*, -C(CH_3_)_3_), 1.43 (6H, *d*, *J* = 5.1 Hz, -CH_3_), 2.50 (1H, *m*, C6-H), 3.16 (1H, *m*, C2-H), 3.52 (4H, *m*, C3′-H, C1′-H), 4.14 (1H, *m*, C2′-H), 5.83 (1H, *s*, -OH), 6.88 (4H, *m*, Ar-H), 7.18 (6H, *m*, Ar-H), 8.39 (1H, *br.s*, -NH_2_^+^), 9.04 (1H, *br.s*, -NH_2_^+^); ^13^CNMR: δ 11.8, 24.9, 42.8, 44.5, 47.3, 47.6, 48.5, 55.6, 56.2, 63.7, 127.4, 128.0, 129.3, 133.2, 141.3, 175.7, 176.3, 199.4; HRMS (*m*/*z*): calculated value for [M + H^+^] 487.2598; found 487.2597.

***4-[3-(diethylamino)-2-hydroxypropyl]-1,7-dimethyl-8,9-diphenyl-4-azatricyclo[5.2.1.0^2,6^]dec-8-ene-3,5,10-trione (*1d*)***

M.W. = 486.6019 (free amine); C_30_H_34_N_2_O_4_*HCl; Yield: 72%; white powder, m.p. 177–179 °C (from MeOH/(Et)_2_O); ^1^HNMR (300 MHz, DMSO-d_6_+ TMS, δ/ppm): 1.18 (6H, *t*, *J* = 7.2 Hz, -CH_3_), 1.42 (6H, *d*, *J* = 4.2 Hz, -CH_3_), 3.16 (6H, *m*, C6-H, C2-H, C3′-H, C1′-H), 3.52 (2H, *m*, -CH_2_-), 3.73 (2H, m, -CH2-), 4.19 (1H, *s*, -OH), 6.87 (4H, *m*, Ar-H), 7.17 (6H, *m*, Ar-H), 9.87 (1H, *br.s*, -NH^+^); ^13^CNMR: δ 8.3, 11.8, 42.8, 46.7, 47.4, 47.6, 54.0, 55.7, 62.5, 127.5, 128.0, 129.3, 133.1, 141.3, 175.8, 176.2, 199.3; HRMS (*m*/*z*): calculated value for [M + H^+^] 487.2598; found 487.2597.

***4-[3-(dibutylamino)-2-hydroxypropyl]-1,7-dimethyl-8,9-diphenyl-4-azatricyclo [5.2.1.0^2,6^]dec-8-ene-3,5,10-trione (*1e*)***

M.W. = 542.7082 (free amine); C_34_H_42_N_2_O_4_*HCl; Yield: 74%; white powder, m.p. 192–196 °C (from MeOH/(Et)_2_O); ^1^HNMR (300 MHz, DMSO-d_6_+ TMS, δ/ppm): 0.89 (6H, *t*, *J* = 7.35 Hz, -CH_3_), 1.27 (4H, *m*, -CH_2_-), 1.42 (6H, *d*, *J* = 4.2 Hz, -CH_3_), 1.60 (4H, *m*, -CH_2_-), 3.05 (4H, *m*, -CH_2_-), 3.20 (1H, *m*, C6-H), 3.31 (2H, *m*, C3′-H), 3.51 (3H, *m*, C1′-H, C2′-H), 4.19 (1H, *m*, C2′-H) 5.94 (1H, *s*, -OH), 6.88 (4H, *m*, Ar-H), 7.17 (6H, *m*, Ar-H), 9.81 (1H, *br.s*, -NH^+^); ^13^CNMR: δ 11.8, 13.4, 19.3, 24.5, 47.4, 47.6, 52.2, 52.8, 55.1, 55.7, 62.6, 127.5, 128.0, 129.3, 133.1, 141.3, 175.8, 176.2, 199.2; HRMS (*m*/*z*): calculated value for [M + H^+^] 543.3224; found 543.3223.

***4-[2-hydroxy-3-(piperidin-1-yl)propyl]-1,7-dimethyl-8,9-diphenyl-4-azatricyclo[5.2.1.0^2,6^]dec-8-ene-3,5,10-trione (*1f*)***

M.W. = 498.6126 (free amine); C_31_H_34_N_2_O_4_*HCl; Yield: 45%; white powder, m.p. 170–171 °C (from MeOH/(Et)_2_O); ^1^HNMR (300 MHz, DMSO-d_6_+ TMS, δ/ppm): 1.42 (6H, *m*, -CH_3_), 1.74 (4H, *m*, -CH_2_-pip), 2.50 (3H, m, C6-H, C3′-H), 2.88 (2H, *m*, -C1′-H), 3.32 (7H, *m*, C2-H, -CH_2_-pip), 4.42 (1H, m, C2′-H) 5.97 (1H, *s*, -OH), 6.88 (4H, *m*, Ar-H), 7.18 (6H, *m*, Ar-H), 9.97 (1H, *br.s*, -NH^+^); ^13^CNMR: δ 11.8, 21.2, 22.1, 42.7, 47.6, 52.1, 53.4, 55.6, 58.7, 62.3, 127.5, 128.0, 129.3, 133.1, 141.4, 175.8, 176.1, 199.3; HRMS (*m*/*z*): calculated value for [M + H^+^] 499.2598; found 499.2597.

***4-[-2-hydroxy-3-(morpholin-4-yl)propyl]-1,7-dimethyl-8,9-diphenyl-4-azatricyclo [5.2.1.0^2,6^]dec-8-ene-3,5,10-trione (*1g*)***

M.W. = 500.5854 (free amine); C_30_H_32_N_2_O_5_*HCl; Yield: 54%; white powder, m.p. 210–212 °C (from MeOH/(Et)_2_O); ^1^HNMR (300 MHz, DMSO-d_6_+ TMS, δ/ppm): 1.42 (6H, *m*, -CH_3_), 3.07 (2H, *m*, C2-H, C6-H), 3.38 (6H, *m*, -CH_2_-morpholin), 3.52 (2H, *m*, -CH_2_-morpholin), 3.88 (4H, *m*, C3′-H, C1′-H), 4.30 (1H, *m*, C2′-H), 6.01 (1H, *s*, -OH), 6.87 (4H, *m*, Ar-H), 7.18 (6H, *m*, Ar-H), 10.07 (1H, *br.s*, -NH^+^); ^13^CNMR: δ 11.8, 42.6, 47.5, 51.0, 52.5, 55.6, 58.7, 62.2, 62.9, 127.5, 128.1, 129.3, 133.1, 141.4, 175.8, 176.1, 199.3; HRMS (*m*/*z*): calculated value for [M + H^+^] 501.2384; found 501.2379.

***4-[3-(ethylamino)-2-hydroxypropyl]-1,7-diethyl-8,9-diphenyl-4-azatricyclo [5.2.1.0^2,6^]dec-8-ene-3,5,10-trione (*2b*)***

M.W. = 486.6019 (free amine); C_30_H_34_N_2_O_4_*HCl; Yield: 55%; white powder, m.p. 197–200 °C (from MeOH/(Et)_2_O); ^1^HNMR (300 MHz, DMSO-d_6_+ TMS, δ/ppm): 0.89 (6H, *m*, -CH_3_), 1.18 (3H, *t*, *J* = 7.2 Hz, -CH_3_), 1.87 (2H, *m*, -CH_2_-), 2.11 (2H, *m*, -CH_2_-), 2.80 (1H, *m*, C6-H), 2.88 (2H, *m*, -CH_2_-) 3.06 (1H, *m*, C2-H), 3.41(2H, *m*, C3′-H), 3.54 (2H, *m*, C1′-H), 4.13 (1H, *s*, C2′-H), 5.89 (1H, *br.s*, -OH), 6.87 (4H, *m*, Ar-H), 7.16 (6H, *m*, Ar-H), 8.67 (1H, *br.s*, -NH_2_^+^), 9.10 (1H, *br.s*, -NH_2_^+^); ^13^CNMR: δ 9.0, 10.7, 18.9, 39.5, 42.2, 42.7, 43.6, 43.7, 49.2, 59.2, 63.3, 127.4, 128.0, 129.2, 133.5, 141.7, 141.8, 176.0, 176.4, 199.0; HRMS (*m*/*z*): calculated value for [M + H^+^] 487.6093; found 487.2591.

***4-[3-(tert-butylamino)-2-hydroxypropyl]-1,7-diethyl-8,9-diphenyl-4-azatricyclo [5.2.1.0^2,6^]dec-8-ene-3,5,10-trione (*2c*)***

M.W. = 514.6551 (free amine); C_32_H_38_N_2_O_4_*HCl; Yield: 67%; white powder, m.p. 231–235 °C (from MeOH/(Et)_2_O); ^1^HNMR (300 MHz, DMSO-d_6_+ TMS, δ/ppm): 0.88 (6H, *d*, *J* = 5.1 Hz, -CH_3_), 1.26 (9H, *m*, -C(CH_3_)_3_), 1.87 (2H, *m*, -CH_2_-), 2.09 (2H, m, -CH_2_-), 2.78 (1H, *m*, C6-H), 3.08 (1H, *m*, C2-H), 3.40 (2H, *m*, C3′-H0, 3.69 (2H, *m*, C1′-H), 4.14 (1H, *m*, C2′-H), 5.80 (1H, *s*, -OH), 6.88 (4H, *m*, Ar-H), 7.16 (6H, *m*, Ar-H), 8.36 (1H, *br.s*, -NH_2_^+^), 8.86 (1H, *br.s*, -NH_2_^+^); ^13^CNMR: δ 9.0, 18.8, 24.9, 42.9, 43.6, 44.3, 56.2, 59.2, 59.3, 63.6, 127.4, 128.0, 129.1, 133.5, 133.5, 141.7, 141.8, 175.9, 176.5, 198.9; HRMS (*m*/*z*): calculated value for [M + H^+^] 515.6625; found 515.2904.

***4-[3-(diethylamino)-2-hydroxypropyl]-1,7-diethyl-8,9-diphenyl-4-azatricyclo [5.2.1.0^2,6^]dec-8-ene-3,5,10-trione (*2d*)***

M.W. = 514.6551 (free amine); C_30_H_34_N_2_O_4_*HCl; Yield: 62%; white powder, m.p. 177–180 °C (from MeOH/(Et)_2_O); ^1^HNMR (300 MHz, DMSO-d_6_+ TMS, δ/ppm): 0.88 (6H, *m*, -CH_3_), 1.19 (6H, *t*, *J* = 7.1 Hz, -CH_3_), 2.06 (2H, *m*, -CH_2_-), 2.08 (2H, *m*, -CH_2_-),3.16 (6H, *m*, C6-H, C2-H, C3′-H, C1′-H), 3.51 (4H, *m*, -CH_2_-), 4.20 (1H, m, C2′-H), 5.98 (1H, *br. s*, -OH), 6.88 (4H, *m*, Ar-H), 7.16 (6H, *m*, Ar-H), 9.87 (1H, *br.s*, -NH^+^); ^13^CNMR: δ 9.3, 9.0, 18.8, 42.8, 43.7, 46.7, 47.4, 48.5, 54.0, 59.2, 62.5, 127.4, 128.0, 129.1, 133.5, 141.7, 176.0, 176.4, 199.0; HRMS (*m*/*z*): calculated value for [M + H^+^] 515.6625; found 515.2904.

***4-[3-(dibutylamino)-2-hydroxypropyl]-1,7-diethyl-8,9-diphenyl-4-azatricyclo[5.2.1.0^2,6^]dec-8-ene-3,5,10-trione (*2e*)***

M.W. = 570.7614 (free amine); C_36_H_46_N_2_O_4_*HCl; Yield: 64%; white powder, m.p. 92–110 °C (from MeOH/(Et)_2_O); ^1^H NMR (300 MHz, DMSO-d_6_+ TMS, δ/ppm): 0.88 (12H, *m*, -CH_3_), 1.29 (4H, *m*, -CH_2_-), 1.62 (4H, *m*, -CH_2_-), 1.87 (2H, *m*, -CH_2_-), 2.07 (2H, *m*, -CH_2_-), 3.06 (4H, *m*, -CH_2_-), 3.19 (2H, *m*, C3-H), 3.52 (2H, *m*, C1′-H), 3.70 (2H, *m*, C2-H, C6-H), 4.23 (1H, *m*, C2′-H), 6.88 (4H, *m*, Ar-H), 7.16 (6H, *m*, Ar-H), 9.99 (1H, *br.s*, -NH^+^); ^13^CNMR: δ 9.0, 13.5, 18.9, 19.4, 24.5, 42.8, 43.6, 52.3, 52.7, 55.2, 59.2, 62.6, 127.4, 127.9, 129.1, 133.5, 141.7, 176.0, 176.4, 198.9; HRMS (*m*/*z*): calculated value for [M + H^+^] 571.7688; found 571.3530.

***4-[2-hydroxy-3-(piperidin-1-yl)propyl]-1,7-diethyl-8,9-diphenyl-4-azatricyclo[5.2.1.0^2,6^]dec-8-ene-3,5,10-trione (*2f*)***

M.W. = 526.6658 (free amine); C_33_H_38_N_2_O_4_*HCl; Yield: 55%; white powder, m.p. 190–195 °C (from MeOH/(Et)_2_O); ^1^HNMR (300 MHz, DMSO-d_6_+ TMS, δ/ppm): 0.89 (6H, *m*, -CH_3_), 1.36 (2H, *m*, -CH_2_-), 1.74 (4H, *m*, -CH_2_-, -CH_2_-pip), 1.87 (2H, *m*, -CH_2_-pip), 2.07 (2H, *m*, -CH_2_-pip), 2.90 (4H, *m*, C3′-H, -CH_2_-pip), 3.16 (2H, *m*, -CH_2_-pip), 3.46 (1H, m, C6-H), 3.49 (2H, C1′-H, C2-H), 4.26 (1H, m, C2′-H), 6.87 (4H, *m*, Ar-H), 7.16 (6H, *m*, Ar-H), 10.08 (1H, *br.s*, -NH^+^); ^13^CNMR: δ 9.0, 18.8, 21.2, 22.1, 42.7, 43.7, 52.1, 53.3, 58.8, 59.2, 62.3, 127.4, 128.0, 129.1, 133.5, 141.7, 176.0, 176.3, 199.01; HRMS (*m*/*z*): calculated value for [M + H^+^] 527.6732; found 527.2904.

***4-[-2-hydroxy-3-(morpholin-4-yl)propyl]-1,7-diethyl-8,9-diphenyl-4-azatricyclo[5.2.1.0^2,6^]dec-8-ene-3,5,10-trione (*2g*)***

M.W. = 528.6386 (free amine); C_32_H_36_N_2_O_5_*HCl; Yield: 54%; white powder, m.p. 226–228 °C (from MeOH/(Et)_2_O); ^1^HNMR (300 MHz, DMSO-d_6_+ TMS, δ/ppm): 0.89 (6H, *m*, -CH_3_), 1.87 (2H, *m*, -CH_2_-), 2.06 (2H, *m*, -CH_2_-), 3.1 (3H, *m*, C3-H, C6-H), 3.36 (3H, *m*, -CH_2_-morpholin, C2-H), 3.47 (4H, *m*, -CH_2_-morpholin), 3.89 (4H, *m*, -CH_2_-morpholin, C1′-H), 4.30 (1H, *m*, C2′-H), 6.01 (1H, *br.s*, -OH), 6.87 (4H, *m*, Ar-H), 7.68 (6H, *m*, Ar-H), 10.57 (1H, *br.s*, -NH^+^); ^13^CNMR: δ 9.0, 18.8, 39.5, 43.7, 51.0, 52.5, 58.8, 59.2, 62.1, 62.9, 127.4, 128.0, 129.1, 133.5, 141.7, 176.0, 176.3, 199.0; HRMS (*m*/*z*): calculated value for [M + H^+^] 529.6460; found 529.2697.

***4-[3-(ethylamino)-2-hydroxypropyl]-1,7,8,9-tetraphenyl-4-azatricyclo[5.2.1.0^2,6^]dec-8-ene-3,5-dione (*3b*)***

M.W. = 568.7040 (free amine); C_38_H_36_N_2_O_3_*HCl; Yield: 45%; white powder, m.p. 254–255 °C (from MeOH/(Et)_2_O); ^1^HNMR (300 MHz, DMSO-d_6_+ TMS, δ/ppm): 1.19 (3H, *t*, *J* = 7.2 Hz, -CH_3_), 2.34 (1H, *m*, C10-H), 2.78 (1H, *m*, C10-H), 2.91 (2H, *m*, -CH_2_-) 3.05 (1H, *m*, C2-H), 3.16 (1H, m, C6-H), 3.30 (2H, *m*, C3′-H), 3.46 (1H, *s*, C2′-H), 4.29 (2H, *m*, C1′-H), 5.85 (1H, *s*, -OH), 6.51 (4H, *m*, Ar-H), 6.88 (6H, *m*, Ar-H), 7.32 (6H, *m*, Ar-H), 7.76 (4H, *m*, Ar-H), 8.60 (1H, *br.s*, -NH_2_^+^), 8.94 (1H, *br.s*, -NH_2_^+^); ^13^CNMR: δ 10.7, 42.2, 49.4, 51.9, 63.0, 63.4, 65.1, 126.5, 126.8, 127.2, 127.8, 129.2, 129.5, 134.4, 139.7, 145.1, 176.6, 176.9; HRMS (*m*/*z*): calculated value for [M + H^+^] 569.7119; found 569.2798.

***4-[3-(tert-butylamino)-2-hydroxypropyl]-1,7,8,9-tetraphenyl-4-azatricyclo[5.2.1.0^2,6^]dec-8-ene-3,5-dione (*3c*)***

M.W. = 596.7575 (free amine); C_40_H_40_N_2_O_3_*HCl; Yield: 60%; white powder, m.p. 177–179 °C (from MeOH/(Et)_2_O); ^1^HNMR (300 MHz, DMSO-d_6_+ TMS, δ/ppm): 1.28 (9H, *s*, -C(CH_3_)_3_), 2.36 (1H, *d*, *J* = 8.7 Hz C6-H), 2.76 (1H, *m*, C10-H), 3.06 (1H, *m*, C10-H), 3.27 (1H, *m*, C2-H), 3.35 (4H, *m*, C3′-H, C1′-H), 3.52 (1H, *m*, C2′-H), 4.21 (1H, *s*, -OH), 6.51 (4H, *m*, Ar-H), 6.88 (6H, *m*, Ar-H), 7.31 (6H, *m*, Ar-H), 7.76 (4H, *m*, Ar-H), 8.41 (1H, *br.s*, -NH_2_^+^), 9.06 (1H, *br.s*, -NH_2_^+^); ^13^CNMR: δ 24.9, 42.7, 44.6, 51.7, 52.0, 56.2, 63.0, 63.7, 65.1, 126.4, 126.5, 127.2, 127.8, 129.1, 129.5, 134.4, 139.7, 145.0, 145.1, 176.5, 177.0; HRMS (*m*/*z*): calculated value for [M + H^+^] 597.7651; found 597.3117.

***4-[3-(diethylamino)-2-hydroxypropyl]-1,7,8,9-tetraphenyl-4-azatricyclo[5.2.1.0^2,6^]dec-8-ene-3,5-dione (*3d*)***

M.W. = 596.7575 (free amine); C_40_H_40_N_2_O_3_*HCl; Yield: 62%; white powder, m.p. 240–247 °C (from MeOH/(Et)_2_O); ^1^HNMR (300 MHz, DMSO-d_6_+ TMS, δ/ppm): 1.20 (6H, *t*, *J* = 7.2 Hz, -CH_3_), 2.33 (1H, *d*, *J* = 8.4 Hz, C6-H), 3.16 (6H, *m*, -CH_2_-, -CH_2_- C3′-H, C1′-H), 3.38 (1H, *m*, C2-H), 3.50 (1H, *m*, C2′-H), 4.26 (2H, *m*, C10-H), 5.98 (1H, *s*, -OH), 6.52 (4H, *m*, Ar-H), 6.88 (6H, *m*, Ar-H), 7.31 (6H, *m*, Ar-H), 7.77 (4H, *m*, Ar-H), 9.93 (1H, *br.s*, -NH^+^); ^13^CNMR: δ 8.4, 42.7, 46.7, 47.4, 51.9, 52.0, 54.2, 62.5, 63.0, 65.1, 126.5, 126.8, 127.2, 127.8, 129.2, 129.5, 134.4, 139.7, 145.0, 145.1, 176.6, 177.0; HRMS (*m*/*z*): calculated value for [M + H^+^] 597.7651; found 597.3117.

***4-[3-(dibutylamino)-2-hydroxypropyl]-1,7,8,9-tetraphenyl-4-azatricyclo[5.2.1.0^2,6^]dec-8-ene-3,5-dione (*3e*)***

M.W. = 652.8635 (free amine); C_44_H_48_N_2_O_3_*HCl; Yield: 64%; white powder, m.p. 237–238 °C (from MeOH/(Et)_2_O); ^1^HNMR (300 MHz, DMSO-d_6_+ TMS, δ/ppm): 0.90 (6H, *t*, *J* = 7.2 Hz, -CH_3_), 1.29 (4H, *m*, -CH_2_-), 1.62 (4H, *m*, -CH_2_-), 2.37 (1H, *m*, C6-H), 3.16 (4, *m*, -CH_2_-), 3.34 (8H, *m*, C2-H, C1′-H, C2′-H, C3′-H, C10-H), 5.94 (1H, *s*, -OH), 6.51 (4H, *m*, Ar-H), 6.88 (6H, *m*, Ar-H), 7.31 (6H, *m*, Ar-H), 7.74 (4H, *m*, Ar-H), 9.71 (1H, *br.s*, -NH^+^); ^13^CNMR: δ 13.5, 19.4, 24.6, 48.5, 51.8, 52.0, 52.3, 52.7, 55.4, 62.6, 63.0, 65.1, 126.5, 126.8, 127.3, 127.8, 129.2, 129.5, 134.4, 139.7, 145.0, 145.1, 176.6, 177.0; HRMS (*m*/*z*): calculated value for [M + H^+^] 653.8714; found 653.3743.

***4-[2-hydroxy-3-(piperidin-1-yl)propyl]-1,7,8,9-tetraphenyl-4-azatricyclo[5.2.1.0^2,6^]dec-8-ene-3,5-dione (*3f*)***

M.W. = 608.7679 (free amine); C_41_H_40_N_2_O_3_*HCl; Yield: 54%; white powder, m.p. 268–270 °C (from MeOH/(Et)_2_O); ^1^HNMR (300 MHz, DMSO-d_6_+ TMS, δ/ppm): 1.35 (1H, *m*, C2-H), 1.75 (5H, *m*, -CH_2_-pip., C6-H), 2.36 (1H, *m*, C10-H), 2.88 (2H, m, C1′-H), 2.99 (1H, *m*, C2′-H), 3.40 (9H, *m*, -CH_2_-pip, C3′-H, C10-H), 5.98 (1H, *s*, -OH), 6.51 (6H, *m*, Ar-H), 6.88 (4H, *m*, Ar-H), 7.31 (6H, *m*, Ar-H), 7.74 (4H, *m*, Ar-H), 9.93 (1H, *br.s*, -NH^+^); ^13^CNMR: δ 21.22, 22.1, 42.6, 51.9, 53.4, 59.0, 62.28, 63.0, 65.1, 126.5, 126.7, 127.2, 127.8, 129.1, 129.5, 134.3, 139.7, 144.9, 145.1, 176.6, 176.9; HRMS (*m*/*z*): calculated value for [M + H^+^] 609.7758; found 609.3117.

***4-[-2-hydroxy-3-(morpholin-4-yl)propyl]-1,7,8,9-tetraphenyl-4-azatricyclo[5.2.1.0^2,6^]dec-8-ene-3,5-dione (*3g*)***

M.W. = 610.7407 (free amine); C_40_H_38_N_2_O_3_*HCl; Yield: 64%; white powder, m.p. 276–277 °C (from MeOH/(Et)_2_O); ^1^HNMR (300 MHz, DMSO-d_6_+ TMS, δ/ppm): 2.34 (1H, *m*, C6-H), 3.07 (3H, *m*, -CH_2_-morpholin, C2-H), 3.29 (5H, *m*, -CH_2_-morpholin, C10-H), 3.46 (3H, *m*, C3′-H, C2′-H), 3.73 (3H, *m*, C1′-H, C10-H), 3.92 (2H, *m*, -CH_2_-morpholin), 6.48 (4H, *m*, Ar-H), 6.88 (6H, *m*, Ar-H), 7.31 (6H, *m*, Ar-H), 7.74 (4H, *m*, Ar-H), 10.45 (1H, *br.s*, -NH^+^); ^13^CNMR: δ 21.22.0, 50.7, 51.9, 52.7, 58.9, 61.9, 63.0, 65.0, 126.5, 126.8, 127.2, 127.8, 129.1, 129.5, 145.1, 176.6, 176.9; HRMS (*m*/*z*): calculated value for [M + H^+^] 611.7486; found 611.2904.

#### 4.1.9. Preparation of Acetate Salt of Derivatives **Ic**, **Ie**, **II**

Alkylamine derivatives were obtained according to the methods published previously [9,10]. Next, the appropriate derivative (0.01 mol) was dissolved in acetic acid (60%) and heated to boiling. After cooling, the excess acid was evaporated to give a crystalline precipitate.

Acetate of 4-[2-(piperidin-1-yl)ethyl]-1,7-diethyl-8,9-diphenyl-4-azatricyclo[5.2.1.0^2,6^]-dec-8-ene-3,5,10-trione (Ic*CH_3_COOH)

M.W. = 496.2720 (free amine); C_32_H_36_N_2_O_3_*CH_3_COOH; Yield: 64%; white powder, m.p. 85–89 °C (from CH_3_COOH); ^1^HNMR (300 MHz, CDCl_3_, δ/ppm): 0.99 (6H, *t*, -CH_3_, *J* = 7.5 Hz), 1.46 (2H, *m*, -CH_2_-), 1.62 (4H, *m*, -(CH_2_)_2_-), 2.02 (2H, *m*, -CH_2_-piper), 2.19 (2H, m, -CH_2_-), 2.67 (4H, m, -CH_2_-piper), 3.48 (2H, *m*, C2-H, C6-H), 3.52 (2H, *m*, -CH_2_-piper), 3.74 (2H, *m*, -CH_2_-piper), 6.90 (4H, *m*, Ar-H), 7.14 (6H, *m*, Ar-H); ^13^CNMR: δ 9.0, 18.9, 23.7, 25.2, 36.2, 43.5, 53.8, 55.2, 59.3, 127.4, 127.9, 129.1, 133.5, 141.5, 176.0, 199.8; HRMS (*m*/*z*): calculated value for [M + H^+^] 497.2799; found 498.2799.

Acetate of 4-[2-hydroxy-3-(propan-2-ylamino)propyl]-1,7-diethyl-8,9-diphenyl-4-azatricyclo [5.2.1.0^2,6^]dec-8-ene-3,5,10-trione (Ie * CH_3_COOH)

M.W. = 500.2669 (free amine); C_31_H_36_N_2_O_4_*CH_3_COOH; Yield: 77%; white powder, m.p. 156–158 °C (from CH_3_COOH); ^1^HNMR (300 MHz, CDCl_3_, δ/ppm): 0.89 (6H, *t*, -CH_3_, *J* = 7.5 Hz), 1.24 (6H, *d*, -CH_3_, *J* = 6.3 Hz), 2.00 (2H, *m*, -CH2-), 2.19 (2H, *m*, -CH_2_-), 2.80 (1H, *m*, C1′-H), 2.89 (1H, *m*, C1′-H), 3.11 (1H, *m*, -CH-), 3.57 (3H, *m*, C2′-H, C3′-H), 3.65 (2H, *m*, C2-H, C6-H), 4.17 (1H, *m*, -OH), 6.87 (4H, *m*, Ar-H), 7.17 (6H, *m*, Ar-H), ^13^CNMR: δ 9.0, 18.9, 21.7, 43.5, 48.4, 50.0, 59.2, 65.7, 127.3, 127.9, 129.2, 133.5, 141.7, 172.7, 176.2, 198.9; HRMS (*m*/*z*): calculated value for [M + H^+^] 501.2748; found 501.2748.

Acetate of 4-[2-(dimethylamino)ethyl]-1,7,8,9-tetraphenyl-4-azatricyclo[5.2.1.0^2,6^]dec-8-ene-3,5-dione (II*CH_3_COOH)

M.W. = 538.2614 (free amine); C_37_H_34_N_2_O_2_*CH_3_COOH, Yield: 82%; white powder, m.p. 203–205 °C (from CH_3_COOH); ^1^HNMR (300 MHz, CDCl_3_, δ/ppm): 2.32 (6H, *m*, -CH3), 2.35 (1H, *m*, C10-H), 2.65 (2H, *t*, *J* = 6.3, C1′-H), 3.17 (1H, *m*, C10-H), 3.64 (2H, *t*, *J* = 6.3, C2′-H), 4.18 (2H, *s*, C2-H, C6-H), 6.55 (4H, *m*, Ar-H), 6.92 (6H, *m*, Ar-H), 7.27 (6H, *m*, Ar-H), 7.72 (4H, *m*, Ar-H); ^13^CNMR: δ 21.0, 36.5, 44.8, 51.8, 55.7, 63.0, 65.3, 126.4, 126.7, 127.1, 127.7, 129.1, 129.5, 134.3, 139.8, 144.9, 176.6; HRMS (*m*/*z*): calculated value for [M + H^+^] 539.2693; found 539.2693.

#### 4.1.10. Determination of Lipophilicity by Reversed-Phase Chromatography

The reversed-phase high-performance liquid chromatography (RP-HPLC) analysis was performed on a Merck–Hitachi LaChrom Elite System (Merck, Darmstadt, Germany) with diode array detector L-2455, thermostat L-2300, pump L-2130, and autosampler L-2200 and with the Waters XTerra MS RP-18 (3.5 μm, 150 × 4.6 mm) chromatographic column (Waters, Milford, MA, USA) as the stationary phase. The mobile phase consisted of methanol, water, and 0.1% (*v*/*v*) formic acid and was degassed by the use of the built-in membrane degasser. The methanol gradient grade for HPLC was purchased from Merck (Merck, Darmstadt, Germany) and formic acid from Polish Reagents (Polish Reagents, Gliwice, Poland); double-distilled water was used. Then, 5 μL of methanolic solutions (0.1%; *m*/*v*) of selected samples were applied to the chromatographic column by the use of an autosampler Hitachi L-2200 (LaChrom Elite, Hitachi-Merck, Darmstadt, Germany). The analysis was performed with the flow rate of 0.7 mL min^−1^ in isocratic mode using various concentrations of organic modifier (methanol) in binary polar mobile phases; percentages of methanol in water were 45–70% (%, *v*/*v*) and changed by 5% per step. Chromatograms were detected at 254 nm and the temperature of the column was 25 °C. All experiments were repeated in triplicate, and the final results were taken to be the arithmetic means. Dead time was measured by the use of uracil (Calbiochem. Merck, Darmstadt, Germany). Statistical and regression analyses were performed using Statistica (ver. 13.3 for Windows). The chromatographic lipophilicity parameters ($logk_{W}$) for selected samples were obtained by the extrapolation of the retention parameter logk to pure water, according to Equation (1):

(1) $$logk=logk_{w}-S\cdot \phi ;$$

where $logk_{W}$ is the value of the retention factor of a substance in pure water, S is the slope of the regression curve, and ϕ is the concentration of the organic modifier [33]. The values of logk were calculated based on the raw HPLC data using the formula (2):

(2) $$logk=\log\left(\frac{t_{R}-t_{0}}{t_{0}}\right);$$

where $t_{R}$ is the retention time and $t_{0}$ is the dead retention time (determined for uracil).

### 4.2. Anticancer Activity

#### 4.2.1. Cells and Cytotoxicity Assay

Human umbilical vein endothelial cells (Life Technologies, Carlsbad, CA, USA) were cultured (according to the manufacturer’s instructions) in Medium 200 containing low serum growth supplement; 1 × 10^4^ cells were seeded on each well on a 96-well plate (Nunc, Roskilde, Denmark). The HeLa (human cervix carcinoma), K562, and MOLT-4 (leukemia) cells were cultured in RPMI 1640 medium supplemented with antibiotics and 10% fetal calf serum in a 5% CO_2_–95% air atmosphere; 7 × 10^3^ cells were seeded on each well on a 96-well plate (Nunc). Twenty-four hours later, cells were exposed to the test compounds for additional 48 hours. Stock solutions of test compounds were freshly prepared in DMSO. The final concentrations of the compounds tested in the cell cultures were 2 × 10^−1^, 1 × 10^−1^, 5 × 10^−2^, 1 × 10^−2^, 1 × 10^−3^, and 1 × 10^−4^ mM. The concentration of DMSO in the cell culture medium was 1%. The values of IC_50_ (the concentration of test compound required to reduce the cell survival fraction to 50% of the control) were calculated from dose–response curves and used as a measure of cellular sensitivity to a given treatment. The cytotoxicity of all compounds was determined by the MTT [3-(4,5-dimethylthiazol-2-yl)-2,5-diphenyltetrazolium bromide; Sigma, St. Louis, MO, USA] assay. Briefly, after 24 or 48 h of incubation with drugs, the cells were treated with the MTT reagent, and incubation was continued for 2 h. MTT-formazan crystals were dissolved in 20% SDS and 50% DMF at pH 4.7, and absorbance was read at 570 and 650 nm on a microplate reader FLUOstar Omega (BMG Labtech, Offenburg, Germany). As a control (100% viability), we used cells grown in the presence of the vehicle (1% DMSO) only.

#### 4.2.2. Analysis of Cell Apoptosis by Caspase-3/7 Activity Assay

First, 20 × 10^3^ K562 cells were seeded on each well of a 96-well plate in RPMI 1640 medium supplemented with 10% fetal calf serum and antibiotics. Cells were grown for 24 h at 37 °C and 5% CO_2_. Test dicarboximides were dissolved in DMSO and added to cell culture to the final concentration of 5 × IC_50_. Cells treated with 1% DMSO served as a negative control, while cells incubated with staurosporine (Sigma, St. Louis, MO, USA) were used as a positive control. Cells were exposed to test compounds for 18 h at 37 °C and 5% CO_2_. Subsequently, the activity of caspase 3 and 7 was measured by Apo-ONE^®^ Homogeneous Caspase-3/7 Assay (Promega, Madison, WI, USA) according to the manufacturer’s instructions. Briefly, the cells were lysed and incubated for 1.5 h with a profluorescent substrate for caspase 3 and 7. Next, the fluorescence was read at an excitation wavelength of 485 nm and emission of 520 nm with a FLUOStar Omega microplate reader (BMG-Labtech, Offenburg, Germany).

#### 4.2.3. Gene Expression Analysis with DNA Microarray

The expression of apoptotic genes was studied using predesigned 384-well microfluidic cards (TLDA TaqMan^®^human apoptosis array, Life Technologies, Applied Biosystems, Foster City, CA, USA). The RT-PCR experiments were executed twice, each on three separate TLDA plates. K562 tumor cells were seeded on a six-well plate (Nunc) in an amount of 3 × 10^6^ cells/well and were exposed to the test compounds at a concentration of 5 × IC_50_ for another 18 h. The control cells were exposed to 1% DMSO or 1 μM staurosporine. The total RNA pool was isolated from the cell lysates using TriPure Isolation Reagent (Roche, Basel, Switzerland) according to the manufacturer’s instruction. The RNA quality was checked with a NanoDrop ND-1000 spectrophotometer (Thermo Fisher Scientific, Waltham, MA, USA). Two micrograms of total RNA were reverse transcribed using a High Capacity cDNA Reverse Transcription kit (Applied Biosystems, Life Technologies). The 384-well TLDA (the qRT-PCR based TaqMan low-density array) cards were configured into four identical 96-gene sets consisting of 93 human genes and three endogenous controls: 18S, ACTB, and GAPDH. The primers (TaqMan Gene Expression Assay, Applied Biosystems) were selected to produce amplicons not longer than 100 bp (a mean of 74 bp). Those 93 genes were categorized into multiple classes including intrinsic, extrinsic, regulatory, and execution apoptosis traits. A reaction mixture with a cDNA template (100 ng) and an equal volume of TaqMan^®^ universal master mix (Applied Biosystems) was immediately loaded into each line of the TLDA microfluidic card. Each card was spun twice (each time for 1 min) at 1200 rpm, then was sealed and loaded into an ABI 7900HT fast real-time PCR system (Applied Biosystems, Waltham, MA, USA), operating for 2 min at 50 °C, 10 min at 94.5 °C, 30 s at 97 °C, and 1 min at 59.7 °C (40 cycles). The results were statistically analyzed using the Student’s *t*-test, assuming a level of significance value of *p* < 0.05. The MetaCoreTM software (Thomson Reuters, from GeneGo) was used to perform the pathway analysis of the differentially expressed genes.

#### 4.2.4. MAPKs Immunoblotting

First, 2 × 10^6^ K562 cells per well were seeded on a six-well plate in the complete medium (RPMI 1640 + 10% FBS). Then, 20 µM JNK kinase inhibitor SP600125 or 10 µM p38 inhibitor SB203580 (Cell Signaling Technology, Danvers, MA, USA) was added for 24 h. Alternatively, K562 cells were exposed to 1% DMSO (control sample), test compound **Ic** (6 µM), **II** (3 µM), or staurosporine (1 µM) for 18 h. Cells exposed to staurosporine served as a positive control in ERK1/2 experiments. K562 cells treated with 20 µM anisomycin (BioShop, Burlington, ON, Canada) for 30 min served as a positive control in JNK and p38 experiments. After incubation, cells were centrifuged (600 rpm, 6 min, 24 °C), washed once with PBS, and lysed in the buffer containing 20 mM Tris (pH 7.5), 150 mM NaCl, and 1% Triton X-100, containing protease (complete protease inhibitor cocktail, Roche) and phosphatase inhibitors (PhosSTOP phosphatase inhibitor cocktail, Roche). Cells were lysed for 10 min on ice and centrifuged (12,000 rpm, 10 min, 4 °C), and supernatants were collected. Total protein concentration in cell lysates was measured with DC Protein Assay (Bio-Rad, Hercules, CA, USA). Cell lysates containing 25 µg of total protein were mixed with Laemli buffer supplemented with SDS and β-Mercaptoethanol, denatured (10 min, 95 °C), and subjected to SDS-PAGE electrophoresis (4% stacking and 10% resolving gels). Resolved proteins were electrotransferred (semi-dry, 90 min, 1.5 mA/cm^2^) to nitrocellulose membranes (0.45 µm, ThermoScientific). Membranes were blocked for 2 h in TBST buffer (20 mM Tris-HCl pH 7.5, 0.9% NaCl, 0.1% Tween 20) containing 5% BSA (BioShop) and probed with primary antibodies (overnight at 4 °C) diluted in TBST buffer with 0.5% BSA. Tubulin served as a loading control for IB experiments. Anti-phospho ERK1/2 (rabbit polyclonal, dilution 1:1000) and anti-ERK1/2 (rabbit polyclonal, dilution 1:1000) were from Cell Signaling Technology. Anti-phospho JNK (mouse polyclonal, dilution 1:250) and anti-JNK (mouse polyclonal, dilution 1:250) were from Santa Cruz Biotechnology (Dallas, TX, USA). Anti-phospho p38 (mouse polyclonal, dilution 1:250) and anti-p38 (mouse polyclonal, dilution 1:250) were from BD Transduction Laboratories (San Diego, CA, USA). Anti-α-tubulin (mouse monoclonal IgG, 1:2000) was from Sigma. After extensive washing with the TBST buffer, membranes were probed with secondary goat anti-rabbit (1:5000 in TBST buffer) or goat anti-mouse HRP-conjugated IgG (1:5000 in TBST buffer, from Santa Cruz Biotechnology) for 45 min at room temperature. Membranes were washed in the TBST buffer, and the chemiluminescent signal was developed with ECL Plus Western Blotting Substrate (Thermo Scientific, Waltham, MA, USA) and visualized in a Syngene G:Box detection system.

#### 4.2.5. Digestion of Plasmid DNA with BamHI Restriction Nuclease

First, 0.5 μg of plasmid DNA (pcDNAHisC, total length 5.5 kbp) containing a unique *BamHI* restriction site was dissolved in a 1× *BamHI* reaction buffer and incubated overnight at 37 °C with the test compounds or daunorubicin. Daunorubicin, a strong intercalating agent, was used as a positive control. The concentration of the test compounds and daunorubicin samples was 100 µM. In the next step, the reaction mixtures were digested with *BamHI* restriction endonuclease (2 U/μL) for 3 h at 37 °C. The total reaction volume was 10 μL. Products of the reaction were subjected to the 1% agarose gel electrophoresis in TBE buffer. The gel was stained with ethidium bromide, and DNA fragments were visualized under a UV lamp (GBox, Syngene).

## Figures and Tables

**Table 1:** Calculated (*clogP*) and experimental (*logk_w_*) parameters of lipophilicity for synthesized compounds. nd—not determined.

Imide Group	Compound	*clogP* (Calculated)	Median *clogP*	*logk_w_* (Experimental)	Median *logk_w_*
2	**Ia**	3.82		4.94	
	**Ib**	4.63		5.09	
	**Ic**	4.95	4.27	5.02	5.05
	**Id**	4.27		5.05	
	**Ie**	4.06		5.17	
1	**1b**	2.48		4.78	
	**1c**	3.22		4.91	
	**1d**	3.15		4.87	
	**1e**	4.97		nd	
	**1f**	3.47		nd	
	**1g**	2.31	3.19	4.83	4.85
	**4**	2.69		nd	
	**1h**	3.84		nd	
	**1j**	2.88		nd	
	**1i**	4.04		nd	
2	**2b**	3.39		5.08	
	**2c**	4.13		4.74	
	**2d**	4.06		5.10	
	**2e**	5.88	4.1	nd	5.03
	**2f**	4.38		nd	
	**2g**	3.22		4.98	
3	**II**	5.45		6.34	
3	**3b**	5.02		6.31	
	**3c**	5.76		6.06	
	**3d**	5.69		6.28	
	**3e**	7.51	5.69	nd	6.28
	**3f**	6.01		nd	
	**3g**	4.85		6.28	
	**5**	5.23		nd	

**Table 2:** The IC_50_ values (µM) for dicarboximides after 48 h incubation with a given cell line. Average values and standard deviations (in parenthesis) from three experiments are shown. Compounds were used as HCl salts except for **Ic** *, **Ie** *, and **II** * which were used as CH_3_COOH salts.

Compound	HeLa	K562	MOLT-4	HUVEC
**1b**	30 (7.5)	32 (8.3)	7 (1.8)	20 (2.4)
**1c**	26 (5.2)	28 (6.7)	28 (5)	23 (3.2)
**1d**	59 (8.9)	39 (9.4)	37 (4.4)	22 (3.3)
**1e**	3.2 (0.4)	5.8 (1.0)	8 (0.9)	30 (4.2)
**1f**	55 (17.6)	18 (3.8)	40 (10.8)	21 (1.6)
**1g**	>100	>100	>100	nd
**4**	61 (14.8)	105 (15.2)	71 (14.8)	56 (4.8)
**1h**	nd	nd	20 (4.7)	64 (7)
**1j**	>100	>100	>100	nd
**1i**	52 (19.2)	30 (4.1)	15 (1.2)	23 (1.8)
**Ic** *	18 (3.1)	20 (3.4)	15 (3.2)	15 (6.3)
**Ie** *	10 (2.6)	8 (1.7)	15 (2.3)	3.5 (0.2)
**2b**	26 (5.6)	8 (1.5)	14 (0.9)	3.4 (0.3)
**2c**	3.2 (0.8)	4 (0.5)	2.5 (0.1)	3.3 (0.2)
**2d**	18 (4.9)	5.8 (1.2)	14 (2.6)	3.6 (0.2)
**2e**	19 (4.5)	4.1 (0.5)	3.1 (0.2)	1.2 (0.06)
**2f**	5.1 (0.7)	3.9 (0.5)	3.4 (0.3)	3.1 (0.3)
**2g**	23 (3.6)	20 (1.4)	18 (2.5)	17 (1.6)
**II** *	20 (2.4)	16 (4.5)	18 (5)	4 (0.5)
**3b**	>100	>100	>100	nd
**3c**	2 (0.4)	2 (0.6)	2.6 (0.3)	0.4 (0.03)
**3d**	1.9 (0.6)	3.2 (0.4)	2.2 (0.3)	0.3 (0.02)
**3e**	40 (8.4)	6 (1.4)	5.4 (0.5)	7 (0.6)
**3f**	3 (0.9)	3 (0.6)	3 (1)	0.4 (0.02)
**3g**	120 (16)	31 (3.6)	5.2 (0.9)	17 (1.8)
**5**	8 (1)	6.2 (1)	2.1 (0.1)	0.7 (0.08)

* denotes compounds as acetate salts; nd—not determined.

## Data Availability

Not applicable.

## Supplementary Materials

Supplementary Materials are available online at https://www.mdpi.com/article/10.3390/ijms22094318/s1.
