# Supplementary material for: New Succinimides with Potent Anticancer Activity: Synthesis, Activation of Stress Signaling Pathways and Characterization of Apoptosis in Leukemia and Cervical Cancer Cells
Source: Int J Mol Sci. 2021 Apr 21;22(9):4318. doi: 10.3390/ijms22094318 (PMC8122671; doi:10.3390/ijms22094318)
Supplement: Supplementary file 1 [file ijms-22-04318-s001.zip › ijms-1152111-supplementary.pdf]

International Journal of *Molecular Sciences*

MDPI

Supplementary Data

Table S1. Heat map analysis of expression of 93 genes associated with apoptosis in K562 cells treated with compound **Ic** or staurosporine. Total RNA was isolated from K562 cells treated with dicarboximide **Ic** (test sample), staurosporine (positive control) or DMSO (control sample) and analyzed using TaqMan human apoptosis array (Applied Biosystems). The change of a gene expression was calculated as a ratio of expression measured in cells treated with staurosporine or compound **Ic** versus cells treated with DMSO (control cells). Several gene transcripts could not be detected and the change in gene expression was not calculated (#DIV/0!). Endogenous control genes are marked in green.

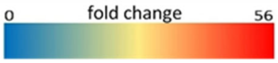

Detector (Gene)	K562 + Staurosporine Ratio	K562 + Compound Ic Ratio
RELB-Hs00232399_m1	0.23	0.40
18S-Hs99999901_s1	0.51	0.80
ACTB-Hs99999903_m1	1.00	1.00
REL-Hs00968436_m1	1.06	0.85
CARD9-Hs00364485_m1	1.36	0.49
RIPK2-Hs01572688_m1	1.57	1.70
NFKB1-Hs00765730_m1	1.65	1.31
BCAP31-Hs01036137_m1	1.89	1.85
CASP7-Hs00169152_m1	1.97	2.78
PEA15-Hs00269428_m1	2.02	1.70
NFKBIB-Hs00182115_m1	2.06	2.29
BCL2L2-Hs00187848_m1	2.14	1.29
BCL3-Hs00180403_m1	2.22	2.30
GAPDH-Hs99999905_m1	2.24	1.56
LRDD-Hs00388035_m1	2.36	2.59
BIRC2-Hs00236911_m1	2.36	1.26
CASP4-Hs01031947_m1	2.62	4.10
HIP1-Hs00193477_m1	2.65	2.04
TNFRSF21-Hs00205419_m1	2.76	2.57
BIRC5-Hs00977611_g1	2.83	2.41
CFLAR-Hs00153439_m1	2.98	2.68
BCL10-Hs00961847_m1	3.07	2.33
NFKBIA-Hs00153283_m1	3.33	3.53
CASP8-Hs01018151_m1	3.49	2.16
MCL1-Hs00172036_m1	3.57	3.59
BBC3-Hs00248075_m1	3.58	4.38
BAD-Hs00188930_m1	3.69	5.30
BID-Hs00609632_m1	3.97	3.28
NALP1-Hs00248187_m1	4.03	3.73
BAK1-Hs00832876_g1	4.13	3.32
BCL2L11-Hs00708019_s1	4.31	4.71
NFKB2-Hs00174517_m1	4.34	4.74
BIRC6-Hs00212288_m1	4.37	3.43
IKBKE-Hs01063858_m1	4.58	2.52
CASP2-Hs00892481_m1	4.76	3.72
BNIP3L-Hs00188949_m1	4.98	5.93

BIRC4-Hs00745222_s1	5.22	7.48
BAX-Hs00751844_s1	5.25	6.08
CARD6-Hs00261581_m1	5.35	2.92
NFKBIZ-Hs00230071_m1	5.41	7.22
BCL2L1-Hs00169141_m1	6.06	4.29
DIABLO-Hs00219876_m1	6.36	3.85
APAF1-Hs00559441_m1	6.43	5.91
BCL2-Hs00608023_m1	6.72	3.42
TNFRSF10A-Hs00269492_m1	6.81	4.16
DAPK1-Hs00234480_m1	7.03	7.39
ESRRBL1-Hs00215973_m1	7.11	5.25
DEDD2-Hs00370206_m1	7.36	8.19
CARD4-Hs00196075_m1	8.14	6.35
IKBKB-Hs00395088_m1	9.28	9.34
TBK1-Hs00179410_m1	9.37	8.92
BNIP3-Hs00969291_m1	9.40	5.59
IKBKG-Hs00175318_m1	9.93	11.64
CRADD-Hs01011159_g1	10.32	11.02
CASP3-Hs00263337_m1	10.52	8.83
CASP10-Hs01017902_m1	11.18	9.25
DEDD-Hs00172768_m1	11.44	15.59
RELA-Hs00153294_m1	12.89	11.35
TNFRSF10B-Hs00366272_m1	12.92	10.82
PMAIP1-Hs00560402_m1	13.67	11.82
CASP6-Hs00154250_m1	13.77	18.16
TNFRSF1B-Hs00153550_m1	18.06	20.82
RIPK1-Hs00169407_m1	20.26	22.20
BCL2L13-Hs00209789_m1	21.99	18.78
CHUK-Hs00989502_m1	26.13	23.80
CASP8AP2-Hs01594281_m1	31.31	28.37
HTRA2-Hs00376860_g1	39.59	51.47
TNFRSF1A-Hs01042313_m1	45.47	54.52
CASP1-Hs00354836_m1	#DIV/0!	#DIV/0!
CASP5-Hs00362072_m1	#DIV/0!	#DIV/0!
CASP9-Hs00154260_m1	#DIV/0!	#DIV/0!
LTB-Hs00242739_m1	#DIV/0!	#DIV/0!
NFKBIE-Hs00234431_m1	#DIV/0!	#DIV/0!
CASP14-Hs00201637_m1	#DIV/0!	#DIV/0!
CARD15-Hs00223394_m1	#DIV/0!	#DIV/0!
BCL2L14-Hs00373302_m1	#DIV/0!	#DIV/0!
BIRC7-Hs00223384_m1	#DIV/0!	#DIV/0!
BIRC8-Hs01057786_s1	#DIV/0!	#DIV/0!
BIRC3-Hs00985031_g1	#DIV/0!	#DIV/0!
FAS-Hs00236330_m1	#DIV/0!	#DIV/0!
FASLG-Hs00181225_m1	#DIV/0!	#DIV/0!
BCL2A1-Hs00187845_m1	#DIV/0!	#DIV/0!
BIK-Hs00154189_m1	#DIV/0!	#DIV/0!
BOK-Hs00261296_m1	#DIV/0!	#DIV/0!
BIRC1-Hs01847653_s1	#DIV/0!	#DIV/0!

TNF-Hs00174128_m1	#DIV/0!	#DIV/0!
TRADD-Hs00601065_g1	#DIV/0!	#DIV/0!
HRK-Hs00705213_s1	#DIV/0!	#DIV/0!
TNFSF10-Hs00234356_m1	#DIV/0!	#DIV/0!
FADD-Hs00538709_m1	#DIV/0!	#DIV/0!
BCL2L10-Hs00368095_m1	#DIV/0!	#DIV/0!
PYCARD-Hs00203118_m1	#DIV/0!	#DIV/0!
ICEBERG-Hs01043258_m1	#DIV/0!	#DIV/0!
TA-NFKBH-Hs01076336_m1	#DIV/0!	#DIV/0!
LTA-Hs99999086_m1	#DIV/0!	#DIV/0!
TNFRSF25-Hs00980365_g1	#DIV/0!	#DIV/0!

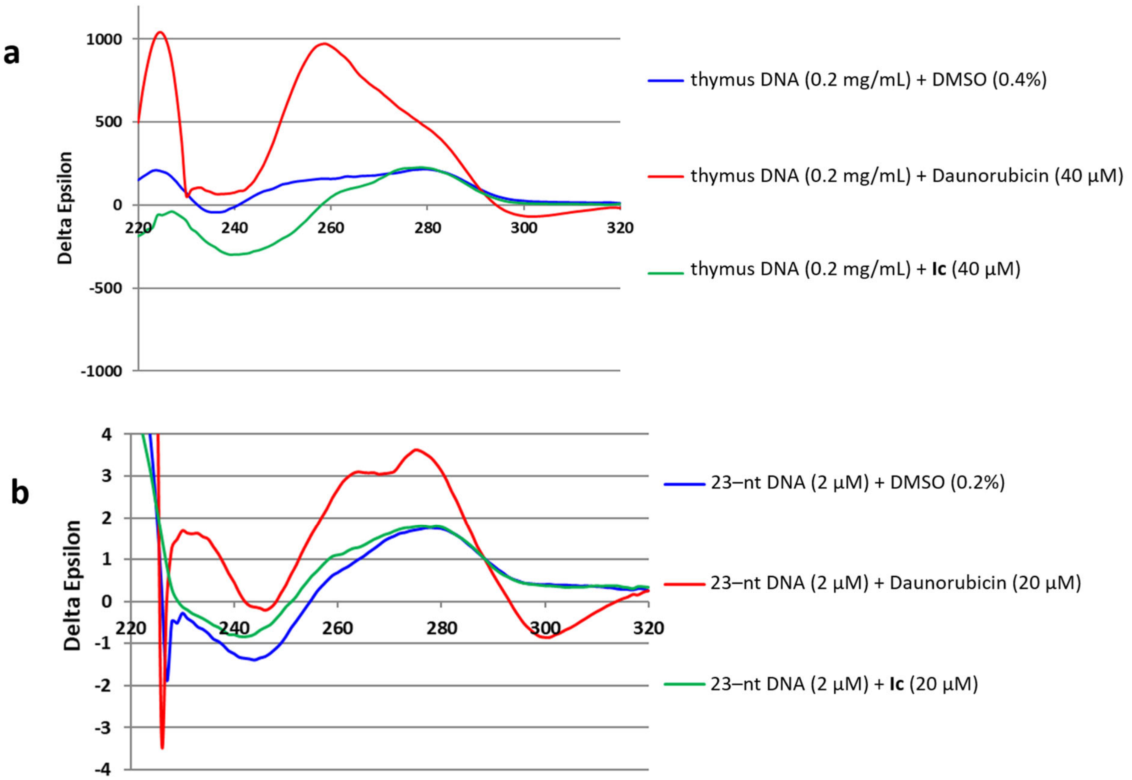

Figure S1. The effect of daunorubicin and compound **Ic** on DNA conformation measured by circular dichroism spectroscopy. Samples for CD analysis were prepared in PBS buffer (pH 7.4) and contained 0.2 mg/mL of thymus DNA (**a**) or 2 µM of the synthetic duplex DNA (**b**). The sequence of synthetic DNA is: 5′TCT TCA AGA ATT CAG GTC CTG AT 3′ (complementary strand is not shown). Test compounds were dissolved in DMSO and added to DNA samples to the concentration of 20 µM (for synthetic duplex DNA) or 40 µM (for thymus DNA). Control samples (blue) contained 0.2% or 0.4% DMSO in the synthetic duplex DNA or thymus DNA samples, respectively. Daunorubicin (red) was used as a reference compound. CD spectra (in the range of 220 – 320 nm) were recorded in the nitrogen atmosphere at 23degC in a quartz cuvette using CD6 spectrometer (Jobin-Yvon, France).

Determination of the chromatographic parameter of lipophilicity, $logk_W$

The chromatographic lipophilicity parameters ($logk_W$) for selected samples were obtained by the extrapolation of the retention parameter $logk$ to pure water, according to Equation:

$logk = logk_w - S \cdot \phi$ (1)

where $logk_W$ is the value of the retention factor of a substance in pure water, S is the slope of the regression curve, and ϕ is the concentration of the organic modifier. The values of $logk$ were calculated based on the raw HPLC data using the formula:

$logk = \log\left(\frac{t_R - t_0}{t_0}\right)$ (2)

where t_R is the retention time and t_0 is the dead retention time (determined for uracil). The goodness of fit of the equation (1) to the experimental data was classified according to Jaffe (Jaffe, H. H. A Reexamination of the Hammet Equation. Chem. Rev. 1953, 53, 191–261). In our RP-HPLC experiments, the goodness of fit of the equation to the experimental data are excellent in most cases (r > 0.99). The F statistic values are, in all cases, higher than the F critical, which confirms the goodness of fit of linear retention-eluent composition equation to experimental data in examined RP-HPLC system. Summarizing, the equation (1) can be used for determination of lipophilicity of investigated compounds.

Table S2. The experimental determination of lipophilicity ($logk_w$) and statistical parameters of linear equation (1) for tested compounds.

Compound	$logk_w$	−S	r	n	F	SD of Estimation
Ia	4.9430	8.1102	0.9972	4	352.1	0.048
Ib	5.0889	8.2610	0.9977	4	426.59	0.045
Ic	5.0243	8.1518	0.9970	4	333.98	0.049
Id	5.0532	8.2849	0.9973	4	365.0	0.049
Ie	5.1736	8.3448	0.9979	4	481.49	0.043
1b	4.7844	8.8320	0.9976	4	407.69	0.049
1c	4.9077	8.8292	0.9980	4	482.93	0.045
1d	4.8734	8.9437	0.9967	4	301.0	0.058
1g	4.8345	8.9729	0.9962	4	262.79	0.062
2b	5.0846	8.2459	0.9970	4	336.0	0.050
2c	4.7442	7.5587	0.9882	4	83.0	0.093
2d	5.1042	8.2603	0.9972	4	359.5	0.049
2g	4.9763	8.1172	0.9977	4	434.58	0.044
II	6.3438	9.2418	0.9972	4	359.8	0.054
3b	6.3071	9.1363	0.9974	4	378.36	0.053
3c	6.0611	8.6589	0.9964	4	275.8	0.058
3d	6.2845	9.0736	0.9976	4	414.31	0.050
3g	6.2759	9.0932	0.9977	4	439.40	0.049
